# Depolarization of the conductance-voltage relationship in the Na_V_1.5 mutant, E1784K, is due to altered fast inactivation

**DOI:** 10.1371/journal.pone.0184605

**Published:** 2017-09-12

**Authors:** Colin H. Peters, Alec Yu, Wandi Zhu, Jonathan R. Silva, Peter C. Ruben

**Affiliations:** 1 Department of Biomedical Physiology and Kinesiology, Simon Fraser University, Burnaby, British Columbia, Canada; 2 Department of Biomedical Engineering, Washington University in St. Louis, St. Louis, Missouri, United States of America; Indiana University School of Medicine, UNITED STATES

## Abstract

E1784K is the most common mixed long QT syndrome/Brugada syndrome mutant in the cardiac voltage-gated sodium channel Na_V_1.5. E1784K shifts the midpoint of the channel conductance-voltage relationship to more depolarized membrane potentials and accelerates the rate of channel fast inactivation. The depolarizing shift in the midpoint of the conductance curve in E1784K is exacerbated by low extracellular pH. We tested whether the E1784K mutant shifts the channel conductance curve to more depolarized membrane potentials by affecting the channel voltage-sensors. We measured ionic currents and gating currents at pH 7.4 and pH 6.0 in *Xenopus laevis* oocytes. Contrary to our expectation, the movement of gating charges is shifted to more hyperpolarized membrane potentials by E1784K. Voltage-clamp fluorimetry experiments show that this gating charge shift is due to the movement of the DIVS4 voltage-sensor being shifted to more hyperpolarized membrane potentials. Using a model and experiments on fast inactivation-deficient channels, we show that changes to the rate and voltage-dependence of fast inactivation are sufficient to shift the conductance curve in E1784K. Our results localize the effects of E1784K to DIVS4, and provide novel insight into the role of the DIV-VSD in regulating the voltage-dependencies of activation and fast inactivation.

## Introduction

Mammalian voltage-gated sodium channels are composed of a single transcript encoding 4 domains (DI-DIV), each with 6 transmembrane segments (S1-S6). The first 4 transmembrane segments of each domain form a voltage-sensing domain (VSD), whereas S5, S6, and the extracellular linker between these segments form the channel pore [1]. In response to membrane depolarization, the positively charged S4 segments rotate and move towards the outside of the membrane, preceding channel pore opening. Outward S4 movements produce a small but measurable gating current that can be used to map the conformational changes of the voltage sensors *en masse* [2]. The S4 segments can also be tethered to fluorophores to measure the movement of individual voltage-sensors [3]. The open sodium channel passes an, inward sodium current which initiates action potentials in neurons, skeletal muscle cells, and cardiac myocytes [4]. Following activation, the DIII-DIV linker segment binds to, and occludes, the internal channel pore, thereby blocking the inward movement of sodium ions [5,6], a process termed fast inactivation. Channels also undergo slow inactivation, during which continued structural rearrangement following prolonged or repetitive depolarizations puts the channels into a non-conducting state [7–9].

Mutations in the genes encoding sodium channels may affect activation, fast inactivation, and/or slow inactivation. Altered sodium channel gating underlies diseases involving neurons, skeletal muscle, and the heart. Mutants in the cardiac sodium channel, Na_V_1.5, are associated with multiple diseases including both Long QT syndrome type 3 (LQT3) and Brugada syndrome type 1 (BrS1) [10,11]. LQT3 is caused by gain-of-function channel defects causing incomplete inactivation and persistent inward current, prolonging the cardiac action potential [10,12]. Action potential prolongation puts the patient at risk for cardiac arrhythmia [13,14].

In contrast, multiple loss-of-function mechanisms have been proposed for BrS1, and there is no clear consensus on the extent to which each mechanism contributes to arrhythmia [15,16]. The depolarization hypothesis for BrS1 suggests that decreased sodium current slows conduction of electrical signals in the heart, which may be exacerbated by fibrosis and drugs. The decreases in conduction velocity lead to a heterogeneity of depolarization, responsible for the characteristic ST-segment elevation and inverted T-wave in BrS [17,18]. In contrast, the repolarization hypothesis suggests that the decreases in sodium current lead to a less positive phase 0 depolarization in the ventricular action potential. The decrease in phase 0 sodium current may allow for the phase 1 transient outward potassium current (I_K,to_), present in epicardial myocardium, to fully repolarize the outer heart wall prior to the action potential plateau. Early repolarization of the epicardium creates a transmural dispersion of repolarization across the heart wall, which causes the BrS ECG sign [19]. Although not the only gene associated with BrS, SCN5A mutations are present in approximately 20% of all BrS patients, making them the most common associated genetic cause [20,21]; thus, understanding the basis for decreases in sodium current remains important even if the precise mechanism by which it contributes to BrS1 is still controversial.

The E1784K mutant in the Na_V_1.5 cardiac voltage-gated sodium channel is the most commonly diagnosed mutant associated with mixed BrS1 and LQT3 [22]. Patients with this mutant can present with either or both elongated QT intervals and ST segment abnormalities in an ECG [22]. E1784K does not alter channel expression, but rather alters channel gating to increase persistent sodium currents and decrease peak transient sodium currents [22]. E1784K decreases the amount of peak sodium current in Na_V_1.5 by shifting the conductance curve to more depolarized membrane potentials and shifting the voltage-dependence of fast inactivation to more hyperpolarized membrane potentials [22,23]. Additionally, E1784K increases the amount of non-inactivating persistent current [22,23].

Previous studies on E1784K have focused on the prevalence of LQT3 and BrS1 signs in carriers and the biophysical characteristics of E1784K ionic currents [22–25]. Our lab recently added studies on the temperature and pH sensitivity of the E1784K mutant heterologously expressed in Chinese Hamster Ovary cells [16,26]. In wildtype (WT) channels, lowered extracellular pH decreases peak sodium currents and increases the fraction of non-inactivating sodium channels [27,28]. Furthermore, cardiac events are known to occur during ischemic heart disease and exercise and are associated with SIDS and cocaine use [29–33]. Although many of these conditions involve complex changes to the body’s internal chemistry, a common outcome is acidaemia. In particular, ischemia and cocaine use are associated with greater than 1 pH unit decreases in extracellular pH [34–37]. As lowered extracellular, but not intracellular, pH is known to decrease peak sodium currents and increase the fraction of persistent current in WT channels, the effects of low extracellular pH on mutant channels, which already may have decreased peak and increased persistent currents, are important [27,28,38]. We previously showed E1784K has preferential sensitivity to changes in extracellular pH, with an increased depolarizing shift of the conductance curve, increased proton block of peak current, and an increase in the fraction of persistent current at pH 6.0 [16]. It is not known, however, if E1784K affects gating currents or whether the preferential effects of lowered extracellular pH on ionic currents in the mutant are due to preferential effects on gating currents.

In this paper, we report the results of experiments using the cut-open oocyte voltage-clamp procedure. We recorded ionic and gating currents to investigate how Na_V_1.5 gating is altered by the E1784K mutant and by changes to extracellular pH. Our experiments were performed using a ‘background’ C373F mutant to increase channel sensitivity to tetrodotoxin (TTX) and allow for full channel block during gating current recordings [39]. We also measured intracellular pH during some recordings to test whether the changes to the biophysical properties of the channels are due to changes in extracellular pH or concurrent changes to intracellular pH. We originally hypothesized that the depolarizing shift in the channel conductance curve in E1784K, and the greater depolarizing shift of conductance at low pH, are a direct result ofgating charge movement occurring at more depolarized membrane potentials. Our results show that this is not the case; the depolarization of conductance in E1784K occurs in the presence of a hyperpolarized gating charge movement. Using a tetrameric model of the sodium channel, fluorescence recordings of labelled S4 voltage-sensors, and experiments on fast-inactivation deficient channels, we show that the depolarizing shift in the conductance curve is likely due to the mutant-dependent hyperpolarization and acceleration of fast inactivation.

## Materials and methods

### DNA constructs

The C373F construct in pPol1 was used in a previous study from our lab and was generously donated by Dr. Mohamed Chahine (Laval University) [40]. The E1784K point mutant was made by Ziwei Ding (Simon Fraser University) and was then cloned into 10β *E*. *coli* cells (New England Biolabs, Ipswich, MA). The Na_V_1.5 IFM/QQQ mutant is in the pRcCMV plasmid. We used a QuikChange Lightning (Agilent Technologies, Santa Clara, CA) kit to perform site directed mutagenesis and make the IFM/QQQ E1784K construct. The plasmid DNA was purified using a Qiagen Midi-prep kit (Qiagen, Hilden, Germany) and sequenced by Eurofin MWG Operon sequencing service (Eurofins Scientific, Luxembourg). We used NotI (NEB) to linearize both the C373F and C373F/E1784K DNA. The DIII voltage-clamp fluorimetry (VCF) (C373Y/M1296C/Y1977A or C373Y/M1296C/E1784K/Y1977A) and DIV VCF (C373Y/S1618C/Y1977A or C373Y/S1618C/E1784K/Y1977A) constructs are in the pMAX plasmid. VCF constructs were linearized with PacI and then purified with the NucleoSpin Gel and PCR Clean-up kit (Macherey-Nagel, Bethlehem, PA). Transcription was performed using a T7 mMESSAGE mMACHINE high yield capped RNA transcription kit (Ambion Inc., Foster City, CA).

### Oocyte preparation

Female *Xenopus laevis* were obtained from Boreal Northwest (St. Catherines, Canada) and Nasco (Salida, CA). At Simon Fraser University frogs were housed in an AQUANEERING Xenopus Aquatic Housing System (Aquaneering Inc., San Diego, CA). Water temperature was maintained at 16–17°C and frogs were fed either Nasco frog brittle or Boreal *Xenopus* food 3 times a week. At Washington University frogs were also housed in an AQUANEERING Xenopus Aquatic Housing System. Water temperature was maintained at 17–18°C and frogs were fed with Nasco’s frog brittle three times a week. Breeding was not performed at either facility.

All animal surgery and animal care procedures were performed in accordance with the policies of the Canadian Council of Animal Care. The protocols for this study were approved by the Simon Fraser Animal Care Committee (SFU Permit 1207K-07) and the Washington University Institutional Animal Care and Use Committee (Protocol 20150237). All surgery was performed under Tricaine Methanesulfonate (2g/L) anesthesia and confirmed by pinching of the hind leg. All efforts were made to minimize suffering. At Simon Fraser University, euthanasia was confirmed by cervical dislocation and pithing of the brain. At Washington University, both terminal and survival surgeries were performed. In terminal surgeries euthanasia was confirmed by excision of the frog’s heart.

The *X*. *laevis* oocyte preparation has been published previously [27]. For ionic and gating current recordings the β1-subunit was not co-injected. The β1-subunit does not alter proton sensitivity, nor does it alter the effects of the E1784K mutant in our experiments [27], and it increases channel expression and ionic current amplitudes beyond what is controllable. We injected oocytes with 55nL of purified Na_V_1.5 α-subunit RNA. For ionic current recordings, we injected RNA at concentrations between 100 ng/uL and 360 ng/uL. Ionic current experiments were performed within 24-72h after injection. Gating currents were recorded between 96-120h after injecting oocytes with 360 ng/uL RNA. For fluorescence recordings cRNA for mutant human α-subunit Nav1.5 and human β1-subunit (to boost expression for maximal fluorescent signals) were injected at a 1:2 molar ratio into oocytes.

### Data acquisition

Cut-open voltage clamp procedures were as described previously [41,42]. We used a CA-1B amplifier (Dagan Corp., Minneapolis, MN) in the cut-open mode. Data were low pass-filtered at 10kHz, digitized at 50kHz, and recorded using Patchmaster (HEKA Electronik, Lambrecht, Germany). For fluorescence recordings Clampex software (v10; Molecular Devices, Sunnyvale, CA) was used for data acquisition. We permeabilized cells via bottom bath perfusion with intracellular solution supplemented with 0.1% saponin. After a 10 – 60s exposure, saponin-free intracellular solution was washed in.

For fluorescence recordings, the light source for stimulation was a green, high-powered LED (PT-121, Luminus, Sunnyvale CA) controlled by a LED driver (Lumina Power, LDPC-30-6-24VDC). The light was then focused into a liquid light guide with a 45°, 5mm compound parabolic concentrator (Edmund Optics, Barrington, NJ) and the guide was coupled to the microscope via a collimating adapter (EXFO, Quebec City, Canada). A 40× water-immersion objective with a numerical aperture of 0.8 (CFI Plan Fluor, Nikon, Tokyo, Japan) was used. Light measurements were made with a photodiode (PIN-040A; United Detector Technology, San Diego, CA) mounted on an XY axis manipulator (Thorlabs Inc., Newton NJ) at the microscope epifluorescence port. The photodiode was attached to the integrating headstage of a patch-clamp amplifier (Axopatch-200A; Molecular Devices) for low noise amplification of the photocurrent. The fluorescence emission was focused onto the photodiode active area using an achromatic doublet (Thorlabs Inc.) with a focal distance of 25mm. Clampex software (v10; Molecular Devices) was used for data acquisition. Recording temperature was maintained at 19°C with a temperature controller (HCC-100A; Dagan Corporation) [3,43].

For ionic current recordings, the extracellular solution contained (in mM): 96 NaCl, 4 KCl, 1 MgCl_2_, 2 CaCl_2_, and 5 HEPES. For ionic current recordings, the intracellular solution contained (in mM): 9.6 NaCl, 88 KCl, 11 EGTA, 5 HEPES. For gating current recordings, the extracellular solution at pH 7.4 contained (in mM): 117.7 NMDG, 122.3 MES, 10 HEPES, and 2 Ca(OH)_2_; and the extracellular solution at pH 6.0 contained (in mM): 70 NDMG, 170 MES, 10 HEPES, and 2 Ca(OH)_2_. The ratio of NMDG to MES was changed to make solutions at the correct pH of the same osmolarity. For gating current recordings, the intracellular solution contained (in mM): 120 NMDG, 120 MES, 10 HEPES, and 2 EGTA. MES was substituted in place of HEPES for all ionic solutions at or below pH 6.5. HCl and NMDG were used to titrate ionic solutions to the desired pH and MES and NMDG were used for gating current solutions. For fluorescence recordings, the internal recording solution was (mM): 105 NMDG-MES, 10 Na-MES, 20 HEPES, and 2 EGTA, pH 7.4. The external recording solution was (mM): 25 NMDG-MES, 90 Na-MES, 20 HEPES, and 2 Ca-MES_2_, pH 7.4.

Gating currents were recorded after addition of 20uL of 50uM TTX to the 0.5 mL top chamber which yields a final TTX concentration of ≈2μM. Data at low pH is matched in all cells to data at pH 7.4. In gating current experiments, TTX was applied after each replacement of the extracellular solution.

Before fluorescence recordings, oocytes were labeled with 20 µM methanethiosulfonate-carboxytetramethylrhodamine (MTS-TAMRA; Santa Cruz Biotechnology, Dallas, TX) in a depolarizing solution (mM: 110 KCl, 1.5 MgCl2, 0.8 CaCl2, 0.2 EDTA and 10 HEPES, pH 7.1) on ice for 20min.

Intracellular pH recordings were made using a VE-2 amplifier in zero current mode (Alembic Instruments, Montreal, Canada). We filled pipettes with 3M KCl solution, and backfilled with hydrogen ionophore solution. The hydrogen ionophore consisted of 6 wt.-% Hydrogen Ionophore II (Santa Cruz Biotechnology) and <1 wt.-% Potassium tetrakis(4-chloro-phenyl)borate dissolved in o-nitrophenol octyl ether. Immediately prior to recordings, the voltage response of the pH electrode was determined with solutions at pH 7.4 and pH 6.0. The average voltage change per pH unit in the electrodes was 46 ± 1 mV/pH unit.

Capacitance was compensated prior to recordings and leak was subtracted using either a P/4 or P/8 protocol.

### Protocols

To measure the voltage-dependence of sodium conductance through the channel pore, we depolarized channels to voltages between -100mV and +60mV for 20ms from a holding potential of -150mV. Conductance was determined by dividing the peak current by the voltage minus the experimentally observed reversal potential. For comparisons of proton block we compared absolute conductance values while our conductance-voltage relationship comparisons were performed with normalized conductance. Conductance-voltage (GV) relationships were fit by a single Boltzmann function (Eq 1)

$$\frac{Y}{Y_{Max}}=\frac{1}{\left(1+e^{\frac{-z*e*(V_{m}-V_{1/2})}{K*T}}\right)}$$ (1)

where Y is conductance, charge, current, or normalized fluorescence; V_m_ is membrane potential in mV; V_1/2_ is the midpoint in mV; z is apparent valence in *e* (elementary charges); K is the Boltzmann constant in meV·K^-1^; and T is temperature in °K.

The proton block of conductance was measured by fitting conductance from individual cells recorded at pH values between 4.0 and 8.0 to a Hill equation.

To measure steady-steady state fast inactivation we conditioned cells to voltages between -150mV and -10mV for 500ms. After the conditioning pulse, currents were elicited by a depolarizing pulse to -10mV. The peak currents were normalized and plotted versus conditioning potential. Steady-state fast inactivation curves were fit by a single Boltzmann function (Eq 1).

We measured persistent current as the fraction of current remaining at the end of 100ms depolarizations to voltages between -30mV and 0mV. To measure the fraction of channels that failed to inactivate, the persistent current was divided by the peak inward current at the same potential to show relative persistent current. For persistent currents, 5 traces were averaged for each measurement.

We measured fast inactivation recovery with a double pulse protocol; following a 500ms depolarization to 0mV, channels were allowed to recover for varying lengths of time at potentials between -130mV and -70mV after which currents were measured by a pulse to -10mV. Recovery time courses were fit by a double exponential function with the fast time constant being analyzed as the time constant of fast inactivation.

We measured open-state fast inactivation by fitting the decay of macroscopic currents with a single exponential function. Closed-state fast inactivation was measured by depolarizing cells to -70mV or -50mV for varying amounts of time from a holding potential of -150mV. The amount of current remaining was measured during a test pulse to -10mV and the normalized current versus onset time was fit by a double exponential function at -70mV and a single exponential equation at -50mV.

Slow inactivation onset and recovery were measured using the same protocol. Currents were measured during a 5ms pulse to -10mV, followed by a depolarization to +30mV, 0mV, or -30mV for between 500ms and 64s (0.5s, 1s, 2s, 4s, 8s, 16s, 32s, 64s), and a repolarization to -120mV, -90mV, or -80mV for a total of 60s. By measuring slow inactivation recovery and onset at different membrane potentials we determined whether any mutant or proton-dependent effects occur throughout the voltage range. Currents were measured at 10 time points after each depolarization pulse (0.02s, 0.1s, 0.25s, 0.5s, 1s, 2s, 5s, 10s, 20s, 60s). Onset at +30mV, 0mV, and -30mV were matched to repolarizations at -120mV, -80mV, and -90mV, respectively. The currents for a given set of recovery pulses were normalized to the current elicited before the depolarizing pulse. The 20ms recovery pulse was not used in analysis as there was still channels in the fast-inactivated state at that point. Slow inactivation recovery time courses at a given membrane potential were globally fit for all onset durations with a double exponential function where the two time constants were global variables. Slow inactivation onset time courses at a given membrane potential were fit globally for recovery durations of 100ms to 10s with a double exponential where the two time constants were global variables. Using this global fit method allows us to more accurately determine the time constants of recovery and onset as the time constants are determined from 8 recovery time courses or 7 onset time courses at each membrane potential.

Charge-voltage (QV) relationships were measured by 20ms depolarizations to potentials between -150mV and +40mV from a holding potential of -150mV. This was followed by a 20ms hyperpolarization to -150mV. We measured the activation of the S4 segments by integrating the outward gating currents (I_g_on) during the initial depolarization steps to give the amount of charge moved at a given membrane potential (QVon). We fit the resulting QV curve with a Boltzmann function. We fit the decay of the outward gating currents with a single exponential function to determine the rate of S4 segment activation.

We measured the voltage-dependence of gating current deactivation by 20ms repolarizations between 20mV and -150mV after a 20ms depolarization to +50mV. The amount of returning charge was assessed by integrating I_g_off during the repolarizing pulse, plotted versus voltage and fit with a Boltzmann function. Results were similar if gating charge return was measured as I_g_on during a depolarization to +50mV immediately following the repolarization.

We measured the rate of gating charge deactivation with a double pulse protocol. We depolarized cells for 500ms to 0mV and then repolarized the cell to -150mV for varying lengths of time. The amount of charge returned during a given time period was measured by integrating an outward gating current elicited by a return pulse to 0mV immediately following the repolarization. The time course of charge return was fit by a double exponential function.

To record both fluorescence and conductance of the voltage-clamp fluorimetry mutants, we depolarized cells in 20mV increments from a holding potential of -100mV. Depolarizing pulses were preceded by a 100ms long prepulse and 50ms long postpulse at -120mV. The amplitude of the fluorescence signals was determined as the mean of the signal from 5ms after depolarizing voltage pulse to the end of voltage pulse. The magnitude of the signal in fluorescence traces is expressed as ΔF/F_0_ (%), where ΔF is the change in signal amplitude in response to the voltage change and F_0_ is the baseline fluorescence recorded with no voltage change.

### Data analysis

Ionic and gating currents were analyzed using Fitmaster (HEKA) and Igor Pro (Wavemetrics). Fluorescence data were analyzed using Clampfit software (v10; Molecular Devices). To correct photobleaching of the fluorescence probe, fluorescence traces were subtracted by the fluorescence baseline, which was recorded with no change in voltage. The magnitude of fluorescence signals is expressed as ΔF/ F0, where ΔF is the change in fluorescence in response to voltage change and F0 is the magnitude of baseline.

All statistical analysis was performed using JMP statistical software (SAS Institute, Cary, NC). Except for the midpoint of proton block of peak conductance, all comparisons were made using a 2 factor repeated measures analysis of variance where the main factors analyzed were pH (ordinal variable) and mutant (nominal variable). The interaction between the two main factors was used to analyze differences in proton sensitivity. A TUKEY post-hoc test was used for pairwise comparisons of pH. For comparisons of time constants, the log of the time constants was compared. To compare proton block of conductance between C373F and C373F/E1784K channels we used a Student’s t-test. Statistical significance was measured at α < 0.05. For many cases where the same parameter was measured at multiple voltages (e.g. persistent current) only the largest P-value (significant effects) or the smallest P-value (non-significant effects) is stated. All midpoint measurements are means and measurements of error listed are standard error of the mean.

### Modeling

All models were run in python on an iMac computer. Graphing and analysis were done using IGOR Pro. Changes in the voltage were simulated as an exponential time course with time constants experimentally determined by fitting the decay of capacitive transients in ionic current or gating current recordings.

## Results

All data sets, including N values and data at pH 7.0 can be found in the S1 Appendix.

### The conductance curve is shifted to more depolarized membrane potentials and the voltage-dependence of S4 movement is shifted to more hyperpolarized membrane potentials in E1784K

**Fig 1:**
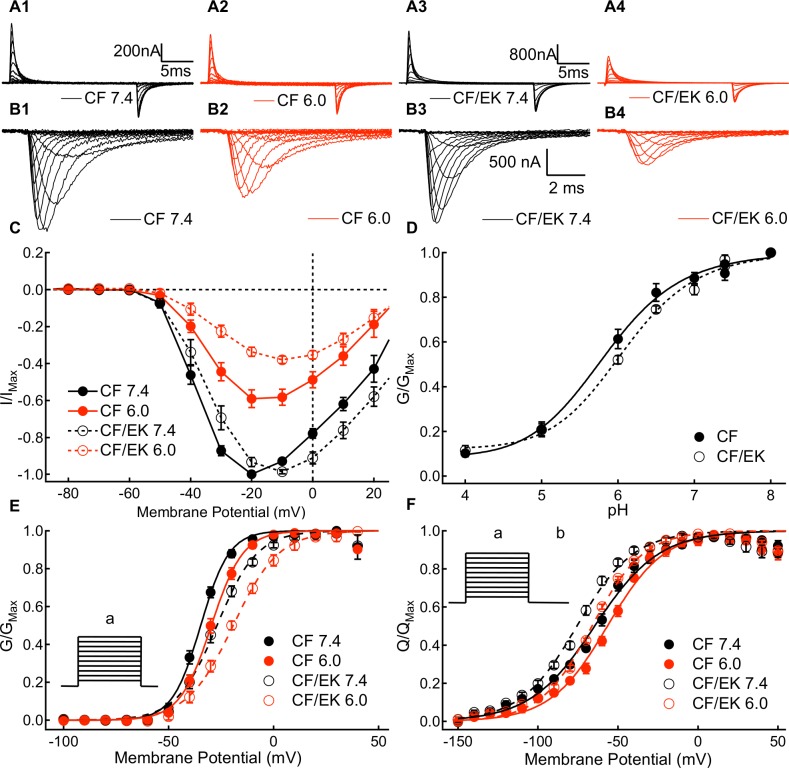
A Depolarizing shift in the C373F/E1784K conductance curve occurs in the presence of a hyperpolarized movement of the voltage-sensors. **(A1-A4)** Sample outward and inward gating currents recorded from C373F and C373F/E1784K Na_V_1.5 at pH 7.4 and pH 6.0. **(B1-B4)** Sample ionic currents recorded from C373F and C373F/E1784K Na_V_1.5 at pH 7.4 and pH 6.0. CF and CF/EK currents with relatively similar amplitudes were selected to highlight the effects of protons. **(C)** Average normalized peak ionic current plotted versus voltage for C373F and C373F/E1784K Na_V_1.5 at pH 7.4 and pH 6.0. Currents at pH 6.0 are normalized to the peak current recorded at pH 7.4 from the same cell. **(D)** Average peak conductance plotted versus pH in C373F and C373F/E1784K Na_V_1.5. Conductance at all pH values is normalized to the peak conductance recorded from the same cell at pH 8.0. The pH at which conductance is reduced by 50% is shifted to higher pH in CF/EK channels. **(E)** Conductance-voltage relationships from ionic currents recorded in C373F and C373F/E1784K Na_V_1.5 at pH 7.4 and pH 6.0. The E1784K mutant shifts the midpoint of the conductance-voltage relationship to more depolarized potentials. E1784K undergoes a larger depolarizing shift of the conductance curve when extracellular pH is lowered. (**E *inset***) To measure current voltage relationships from which conductance was determined, cells were depolarized to membrane potentials between -100mV and +60mV (a) from a holding potential of -150mV. **(F)** Charge-voltage relationships for outward gating currents recorded from C373F and C373F/E1784K Na_V_1.5 at pH 7.4 and pH 6.0. The E1784K mutant shifts the midpoint of the charge-voltage relationship in the hyperpolarizing direction. (**F *inset***) To measure gating current activation, cells were depolarized to membrane potentials between -150mV and +40mV (a) followed by a hyperpolarization to -150mV (b).

We measured the voltage-dependence and rate of S4 voltage sensor movement by recording outward and inward gating currents for CF and CF/EK channels at pH 7.4 and pH 6.0 (Fig 1A1–1A4). Channel conductance was measured by recording macroscopic ionic currents at membrane potentials between -100mV and +60mV (Fig 1B1–1B4).

We measured intracellular pH concurrently in some experiments and found that lowering extracellular pH to pH 6.0 does not significantly affect intracellular pH in uninjected oocytes or those expressing CF or CF/EK channels (S1 Fig) (P = 0.9760).

We compared the dependence of peak currents and conductance in CF and CF/EK channels between pH 4.0 and pH 8.0. The current-voltage relationships for CF and CF/EK channels at pH 6.0 normalized to currents at pH 7.4 are shown in Fig 1C. The maximal conductance at all pH values normalized to the maximal conductance at pH 8.0 in the same cell is shown for CF and CF/EK channels in Fig 1D. There was a significant shift in the pKa of conductance block of CF/EK channels compared to CF channels (P = 0.0149).

We determined the voltage-dependence of sodium channel conductance in CF and CF/EK Na_V_1.5 channels at pH 7.4, 7.0, and 6.0 (Fig 1E and Table A in the S1 Appendix). Both the presence of the E1784K mutant (P = 0.0004) and lowering extracellular pH to pH 7.0 and 6.0 (P = 0.0253 and P < 0.0001, respectively) cause a significant shift of the normalized conductance curve of CF Na_V_1.5 to more depolarized membrane potentials. The depolarizing shift due to decreases in pH is significantly larger in the CF/EK mutant than in CF (P = 0.0374). Both the E1784K mutant (P = 0.0002), and lowering extracellular pH to pH 6.0 from pH 7.4 (P < 0.0001), cause significant decreases in the apparent valence of the conductance curve.

The rate of outward gating charge movement was measured by fitting the decay phase of outward gating currents to a single exponential function (S2 Fig). E1784K does not significantly alter the rate of outward gating charge movement at most measured potentials (P ≥ 0.1368 for 13 of the 15 measured voltages). The total gating charge moved at a given voltage was determined by taking the time integral of the outward gating current during a depolarization step. Normalized outward charge versus voltage relationships were measured for C373F and C373F/E1784K at pH 7.4 and pH 6.0 (Fig 1F and Table G in the S1 Appendix). Decreasing pH from pH 7.4 to pH 6.0 causes a significant shift in the midpoint of the charge-voltage relationship in the depolarized direction in both CF and CF/EK channels (P < 0.0001). Although E1784K causes a depolarizing shift in the midpoint of the conductance curve (Fig 1E and Table A in the S1 Appendix), it causes a hyperpolarizing shift in the midpoint of the charge-voltage relationship (Fig 1F and Table G in the S1 Appendix) (P < 0.0001). Thus, the channel voltage-sensors move outwards at more negative potentials in the mutant, but the channel does not conduct ions until more positive potentials.

Contrary to our hypothesis, these results suggest the basis for the shift in the midpoint of the conductance curve caused by E1784K is not due to a direct effect on the voltage sensors responsible for activation.

### Fast inactivation is accelerated and shifted in the hyperpolarizing direction in E1784K

**Fig 2:**
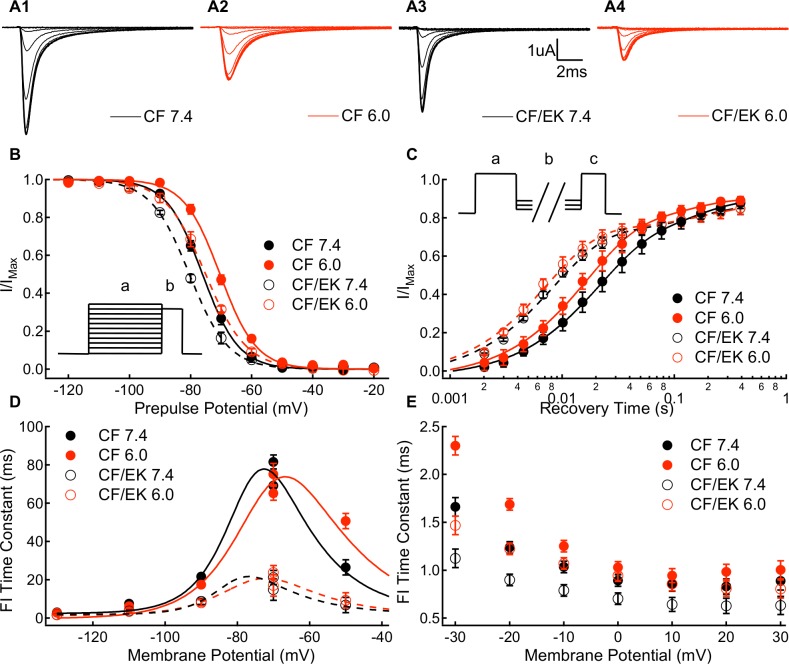
E1784K shifts thevoltage-dependence of fast inactivation to more hyperpolarized potentials and accelerates the rate of onset and recovery. **(A1-A4)** Sample ionic currents elicited during steady-state fast inactivation recordings from C373F and C373F/E1784K Na_V_1.5 at pH 7.4 and pH 6.0. **(B)** Steady-state fast inactivation relationships for C373F and C373F/E1784K Na_V_1.5 at pH 7.4 and pH 6.0. The E1784K mutant shifts the midpoint of the fast inactivation voltage-dependence in the hyperpolarizing direction, whereas decreasing extracellular pH to pH 6.0 shifts the midpoint of the fast inactivation voltage-dependence to more depolarized membrane potentials. (**B *inset***) To measure steady-state fast inactivation, cells were depolarized to -10mV (b) following a conditioning pulse to membrane potentials between -150mV and -10mV (a). **(C)** Recovery from fast inactivation time course at -90mV for C373F and C373F/E1784K Na_V_1.5 at pH 7.4 and pH 6.0. The E1784K mutant accelerates recovery from inactivation. (**C *inset***) To measure recovery from inactivation, cells were depolarized to -10mV (c) following a conditioning pulse to 0mV (a) and a recovery pulse of variable duration to membrane potentials between -130mV and -70mV (b). **(D)** Time constants of fast inactivation recovery (-130mV to -70mV) and closed-state fast inactivation onset (-70mV and -50mV) plotted versus voltage for C373F and C373F/E1784K Na_V_1.5 at pH 7.4 and pH 6.0. The E1784K mutant accelerates fast inactivation recovery and closed-state fast inactivation onset at all potentials. **(E)** Time constants of open-state fast inactivation onset for C373F and C373F/E1784K Na_V_1.5 at pH 7.4 and pH 6.0. The E1784K mutant accelerates open-state inactivation at all membrane potentials excluding +20mV.

We measured the voltage-dependence of fast inactivation in CF and CF/EK Na_V_1.5 channels using a test pulse to -10mV following a 500ms prepulse to membrane potentials between -150mV and -10mV (Fig 2A1–2A4). The normalized currents recorded during the test pulse are plotted versus prepulse potential in Fig 2B (Table A in the S1 Appendix). The E1784K mutant causes a significant hyperpolarizing shift in the midpoint of the voltage-dependence of fast inactivation (P = 0.0003). Decreasing pH to 7.0 or 6.0 from pH 7.4 causes a significant depolarizing shift in the midpoint of the voltage-dependence of fast inactivation (P = 0.0312 and P < 0.0001, respectively). The shift in the voltage-dependence of fast inactivation to more hyperpolarized membrane potentials due to the E1784K mutant parallels the hyperpolarizing shift in the E1784K QV curve (Fig 1F and Table G in the S1 Appendix).

The time course of ionic current recovery from fast inactivation at -90mV is plotted in Fig 2C. The time constants of fast inactivation recovery at voltages between -130mV and -70mV were measured as the faster time constant in bi-exponential fits to data like that found in Fig 2C. Similarly, the time constants of closed-state fast inactivation onset at -70mV and -50mV were measured as the faster time constant in bi-exponential fits to fast inactivation onset time courses. The fast time constants of fast inactivation recovery and closed-state onset are plotted versus membrane potential in Fig 2D (Table B in the S1 Appendix). E1784K significantly decreases the fast time constants of fast inactivation recovery at -130mV, -110mV, -90mV, and -70mV (P < 0.0001 in all cases). E1784K also significantly decreases the time constant of closed-state fast inactivation onset at -70mV and -50mV (P < 0.0001 in both cases). Lower extracellular pH significantly decreases the fast time constant of fast-inactivation recovery at -110mV (P = 0.0002) and -90mV (P = 0.0054) and increases the time constant of closed-state fast inactivation at -50mV (P < 0.0001).

The time constants of open-state fast inactivation were determined by fitting mono-exponential equations to ionic current decay (Table C in the S1 Appendix). The time constants of open-state fast inactivation onset are plotted versus voltage in Fig 2E. E1784K decreases the time constant of open-state inactivation at voltages between -40mV and 30mV, excluding at +20mV (P < 0.044 for all cases, except at +20mV P = 0.0763). Thus, the E1784K mutant accelerates both entry into and recovery from the fast-inactivated state. Lower extracellular pH significantly increases the time constant of open-state fast inactivation between -40mV and +30mV (P < 0.0408).

### Persistent currents are larger and more proton-sensitive in E1784K

**Fig 3:**
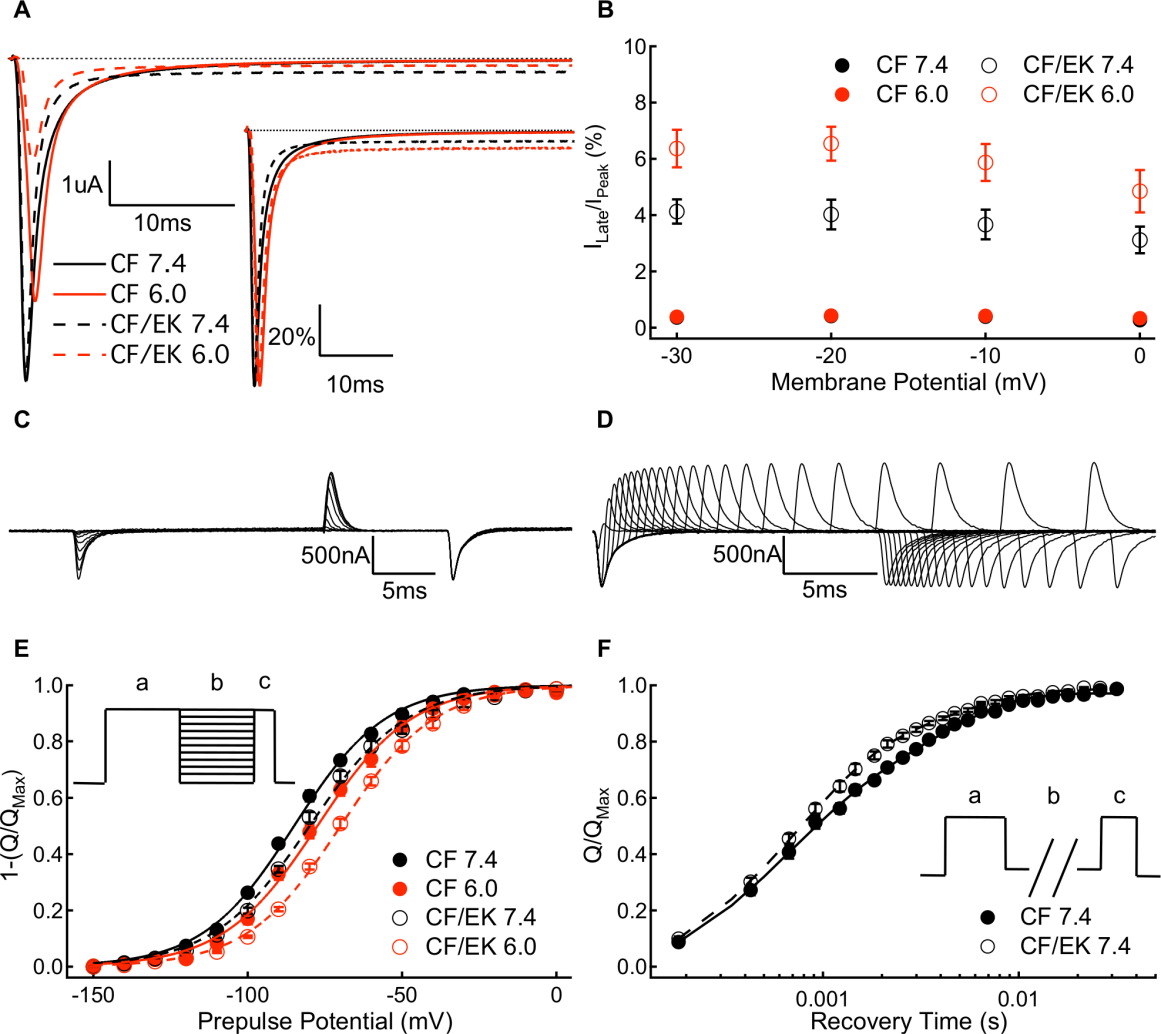
Protons cause a greater increase in persistent sodium current in C373F/E1784K Na_V_1.5. **(A)** Sample persistent sodium current recordings at -20mV from C373F and C373F/E1784K Na_V_1.5 at pH 7.4 and pH 6.0. **(A *inset*)** Sample persistent sodium current recordings at -20mV normalized to peak current from C373F and C373F/E1784K Na_V_1.5 at pH 7.4 and pH 6.0. **(B)** Persistent current normalized to peak current for C373F and C373F/E1784K Na_V_1.5 at pH 7.4 and pH 6.0 at membrane potentials between -30mV and 0mV. The E1784K mutant increases the fraction of persistent current. When extracellular pH is lowered to pH 6.0 there is a larger increase in the fraction of persistent current in CF/EK compared with CF. **(C)** Sample gating currents recorded during a protocol to measure gating current deactivation voltage-dependence. **(D)** Sample gating currents recorded to measure the time course of voltage-sensor deactivation at -150mV. **(E)** Voltage-dependence of gating current deactivation for C373F and C373F/E1784K Na_V_1.5 at pH 7.4 and pH 6.0. The inverse of the amount of charge deactivating at a given voltage is plotted to facilitate comparisons with the outward charge-voltage relationship. The voltage-dependence of gating current deactivation is shifted in the depolarizing direction by the E1784K mutant. (**E *inset***) To measure the voltage-dependence of gating current deactivation, cells were hyperpolarized to membrane potentials between -150mV and +20mV (b) following a depolarization pulse to +50mV (a). This was followed by depolarization to +50mV (c). **(F)** Time course of gating charge deactivation at -150mV for C373F and C373F/E1784K Na_V_1.5 at pH 7.4. Time courses at pH 6.0 overlap those recorded at pH 7.4 and are therefore not shown. The E1784K mutant increases the fraction of charge which recovers with the fast time constant of recovery and decreases the fraction of slow charge return. (**F *inset***) To measure the rate of gating current deactivation, we depolarized cells to 0mV (c) following a conditioning pulse to 0mV (a) and a recovery pulse of variable length to -150mV (b).

We measured absolute (Fig 3A) and relative (Fig 3A *inset*) persistent current amplitudes in CF and CF/EK Na_V_1.5 channels at pH 7.4, 7.0, and 6.0 at membrane potentials between -30mV and 0mV. The CF/EK mutant has a significantly larger proportion of persistent current compared to CF channels at all membrane potentials (Fig 3B and Table D in the S1 Appendix) (P ≤ 0.0018). Low pH significantly increases the relative persistent current more in the CF/EK mutant compared to CF channels at all membrane potentials (P ≤ 0.0002). Thus, whereas CF channels have a relatively similar fraction of non-inactivating current at -20mV at pH 7.4 and pH 6.0 (0.45% and 0.43%, respectively), the CF/EK channels have a 2.5% increase in persistent current from pH 7.4 to pH 6.0 at -20mV (4.0% and 6.5%, respectively). The CF/EK mutant also leads to an increase in the absolute persistent current at all membrane potentials (P ≤ 0.0058).

### The gating current deactivation curve is shifted in the depolarizing direction and accelerated in E1784K

The voltage-dependence of gating current deactivation was measured by taking the time integral of inward gating currents at membrane potentials between +20mV and -150mV after depolarizing pulses to move the S4 segments to their activated conformation (Fig 3C). Similar results were obtained when the amount of charge deactivation was measured by integrating outward gating currents at +50mV immediately after the test pulse. To facilitate comparison with activating gating currents, the inverse of the total inward charge is plotted versus voltage (Fig 3E). Thus, similar to our outward charge-voltage relationships, our deactivation voltage-dependence represents how much gating charge is in the outward conformation. In contrast to the shift in the midpoint of the activation charge-voltage relationship to more hyperpolarized membrane potentials, E1784K causes a significant depolarizing shift in the voltage at which gating charge deactivates (P = 0.0007) (Table G in the S1 Appendix).

We measured the rate of gating charge deactivation at -150mV (Fig 3D). The amount of gating charge that has deactivated for a given recovery time is plotted in Fig 3F and is fit by a bi-exponential equation (Table H in the S1 Appendix). The fast and slow time constants of deactivation were not significantly altered by E1784K (P = 0.0812 and P = 0.1809, respectively). In the CF/EK channels the amplitude of the fast time constant was significantly increased and the amplitude of the slow time constant is significantly decreased compared to CF channels (P = 0.0012 and P = 0.0082, respectively). Thus, the overall rate of gating charge deactivation is accelerated by the E1784K mutant.

### A 10s component of slow inactivation is accelerated in E1784K

**Fig 4:**
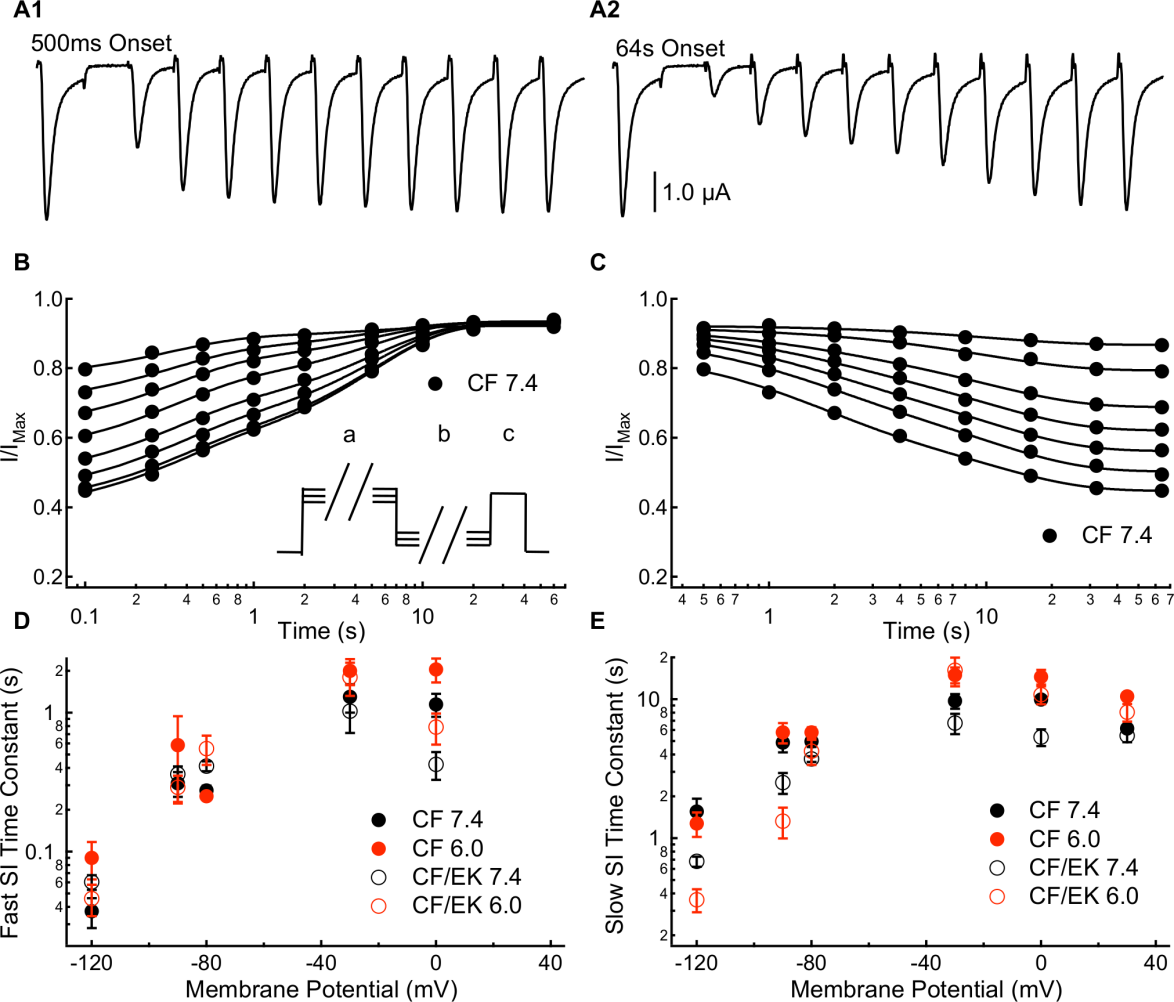
E1784K accelerates a component of slow inactivation with 1-10s time constants. **(A1 and A2)** Sample inward sodium currents recorded after 20ms to 60s repolarization pulses to -90mV following either a 500ms or 64s depolarization to -30mV. **(B)** Time course of slow inactivation recovery at -90mV in C373F Na_V_1.5 at pH 7.4 following depolarizations to -30mV for durations between 500ms (top curve) and 64s (bottom curve). (**B *inset***) To measure onset and recovery time courses for slow inactivation, cells were depolarized to -10mV (c) following a depolarizing pulse of variable duration to membrane potentials between -30mV and +30mV (a) and a recovery pulse of variable duration to membrane potentials between -120mV and -80mV (b). **(C)** Time course of slow inactivation onset at -30mV in C373F Na_V_1.5 at pH 7.4 with recovery interpulses to -90mV for durations between 100ms (bottom curve) and 10s (top curve). **(D)** Time constants for the fast component of slow inactivation recovery (-120mV to -80mV) and slow inactivation onset (-30mV and 0mV) plotted versus voltage for C373F and C373F/E1784K Na_V_1.5 at pH 7.4 and pH 6.0. The E1784K mutant decelerates the fast component of inactivation recovery at -80mV and accelerates inactivation at 0mV. Decreasing extracellular pH slows the fast component of inactivation onset at 0mV. **(E)** Time constants for the slow component of slow inactivation recovery (-120mV to -80mV) and slow inactivation onset (-30mV to 30mV) plotted versus voltage for C373F and C373F/E1784K Na_V_1.5 at pH 7.4 and pH 6.0. The E1784K mutant accelerates the slow component of recovery at -120mV and -90mV and accelerates the slow component of onset at 0mV. Decreasing extracellular pH slows the slow component of inactivation onset at -30mV, 0mV, and +30mV.

Slow inactivation onset and recovery were measured in CF and CF/EK channels using a multiple pulse protocol with slow inactivation onset durations between 500ms and 64s and recovery durations between 20ms and 60s (Fig 4A1 and 4A2). The resulting slow inactivation recovery and onset curves were fit with double exponential functions (Fig 4B and Table E in the S1 Appendix; and Fig 4C and Table F in the S1 Appendix, respectively), except at +30mV where only a single time component of slow inactivation could be fit. The currents recorded after 20ms of recovery were not used in this analysis as many channels were still fast-inactivated at that time, based on the time constants from our fast inactivation recovery experiments (Fig 2D and Table B in S1 Appendix) The fast and slow time constants of slow inactivation recovery and onset are plotted versus voltage in Fig 4D and 4E, respectively.

The recovery of the fast component of slow inactivation is slowed in the CF/EK mutant at -80mV (P = 0.0149) whereas the fast component of slow inactivation onset at 0mV is significantly accelerated in the CF/EK mutant compared to CF (P = 0.0052) (Fig 4D and Tables E and F in the S1 Appendix). The mutant effect on the fast component of slow inactivation recovery was not significant at other voltages (-120mV: P = 0.1168, -90mV: P = 0.7984, -30mV: P = 0.7962) (Fig 4D and Tables E and F in the S1 Appendix). The slow component of slow inactivation recovery and onset is accelerated in E1784K at -120mV, -90mV, and 0mV (P = 0.0017, P = 0.0397, and P = 0.0030) while at -80mV and -30mV a non-significant trend was seen (P = 0.0945, P = 0.0865) (Fig 4E and Tables E and F in the S1 Appendix). In total these data suggest that E1784K primarily accelerates the rate of a component of slow inactivation onset and recovery in the 1-10s range.

Decreasing extracellular pH significantly slows the fast component of slow inactivation onset at 0mV (P = 0.0029) (Fig 4D and Table F in the S1 Appendix) and the slow component of slow inactivation onset at -30mV (P = 0.0169), 0mV (P = 0.0009), and +30mV (P < 0.0001) (Fig 4E and Table F in the S1 Appendix). Decreasing extracellular pH does not significantly affect the fast component of slow inactivation recovery at any membrane potential (P ≥ 0.6884) (Fig 4D and Table E in the S1 Appendix). In contrast, decreasing extracellular pH causes a non-linear effect on the slow component of slow inactivation recovery at -120mV, -90mV, and -80mV. In all cases the slow recovery time constant was slowest at pH 7.0 compared to pH 7.4 and pH 6.0 (Table E in S1 Appendix). The changes to the slow component of recovery were consistent in CF and CF/EK channels. Since time constants reflect both the forward and reverse rates of slow inactivation, it is possible that one rate is affected by a change to pH 7.0 whereas the other is not affected until pH reaches 6.0. Overall, these data suggest that decreases in extracellular pH primarily impacts slow inactivation in the 1-10s range as opposed to the range less than 1s.

### DIVS4 movement is shifted to more hyperpolarized potentials in E1784K

**Fig 5:**
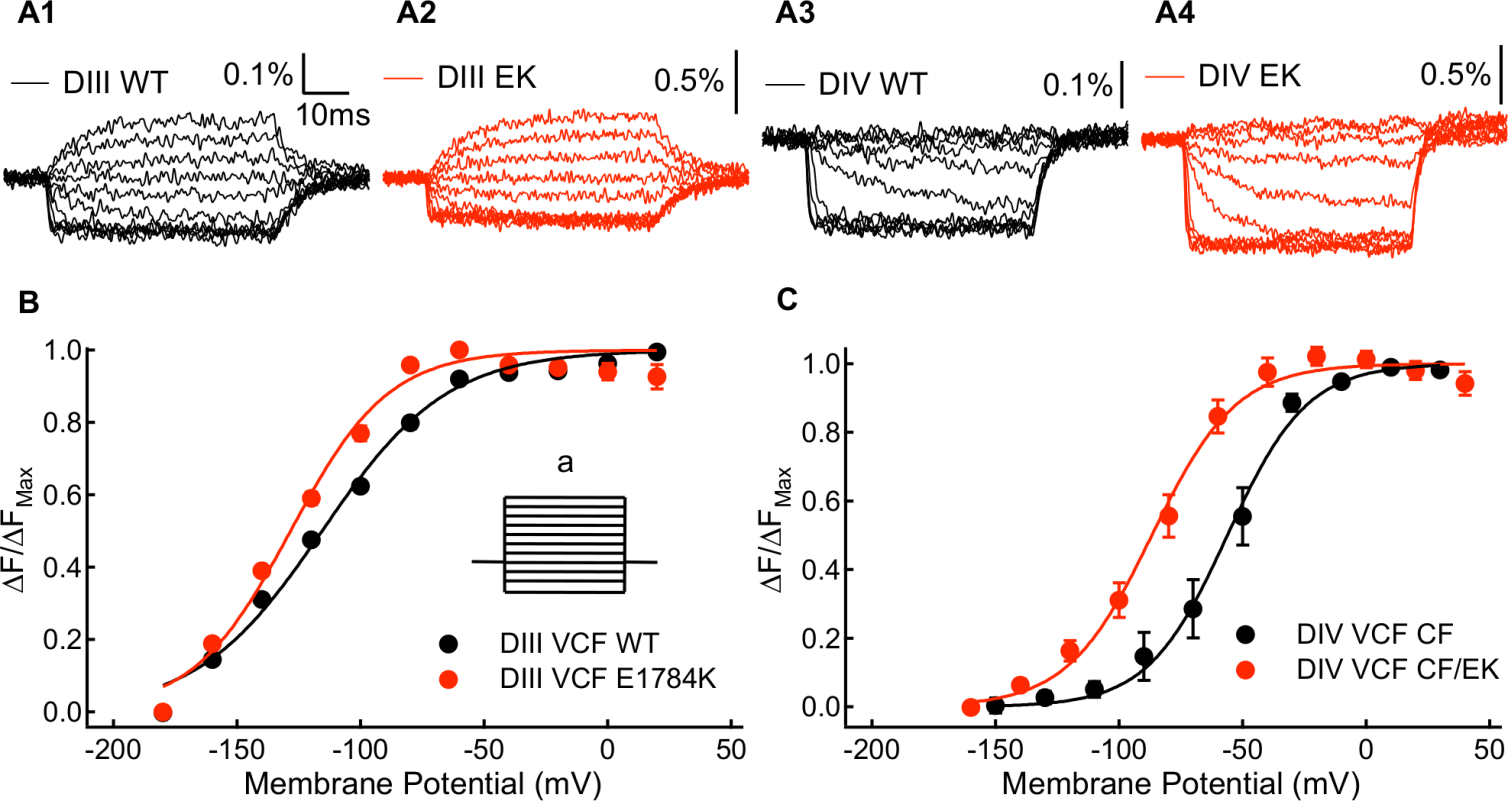
Kinetic and steady-state properties of fluorescence signals from the WT-DIII-VCF, E1784K-DIII-VCF, WT-DIV-VCF, and E1784K-DIV-VCF constructs. (**A1-A4**) Representative fluorescence signals for DIII-VCF, E1784K-DIII-VCF, DIV-VCF, and E1784K DIV-VCF constructs. Percentage of fluorescence change were calculated as ΔF/F0. (**B**) Voltage-dependence of steady state fluorescence signal change (FV) of WT-DIII-VCF and E1784K-DIII-VCF constructs. The E1784K mutant causes a shift in the DIII FV curve to more hyperpolarized membrane potentials. (**B *inset***) To measure the voltage-dependence of voltage-sensor fluorescence, the membrane potential of cells was changed to between -180mV and +20mV (a) from a holding potential of -120mV. (**C**) Voltage-dependence of steady state fluorescence signal change (FV) of WT-DIV-VCF and E1784K-DIV-VCF constructs. The E1784K mutant shifts the DIV FV curve in the hyperpolarizing direction.

From the activating and deactivating gating charge measurements, we observed that E1784K mutant affects channel voltage sensing domains. Based on: (1) the position of the mutant, (2) changes in fast inactivation, (3) increases in persistent current, and (4) relatively smaller fraction of slowly deactivating gating charge, altered movement of the voltage-sensors in DIII and DIV seems likely [44,45]. To confirm whether the DIII and DIV voltage-sensors are affected by E1784K, we used the voltage clamp fluorometry (VCF) method. The VCF constructs used a C373Y mutant instead of a C373F mutant to increase channel sensitivity to TTX. They also contain a Y1977A mutation to prevent ubiquitination of the channel to increase expression level. Sample traces of CY and CY/EK, DIII and DIV voltage sensor fluorescence are shown in Fig 5A1–5A4. E1784K in these two VCF constructs induces similar shifts in the midpoints of the channel conductance and fast inactivation curves (S3 Fig) as in the non-VCF constructs (Figs 1E and 2B). The voltage-dependence of the VSD activation is described by the fluorescence-voltage (FV) curve. Compared to CY channels, CY/EK induces a significant shift in the hyperpolarizing direction in midpoint of the DIV FV (P = 0.0126) (Fig 5C and Table I in the S1 Appendix), suggesting that the DIV-VSD activates at lower potentials in the presence of the E1784K mutant. E1784K also causes a small shift in the hyperpolarizing direction in the midpoint of the DIII F-V curve (P = 0.0193) (Fig 5B and Table I in the S1 Appendix). Since the DIII and DIV VSDs have been shown to closely regulate each other, it is possible that any E1784K modulation of the DIII-VSD is a secondary effect of modifications to the DIV-VSD [46].

### Channel modeling

**Fig 6:**
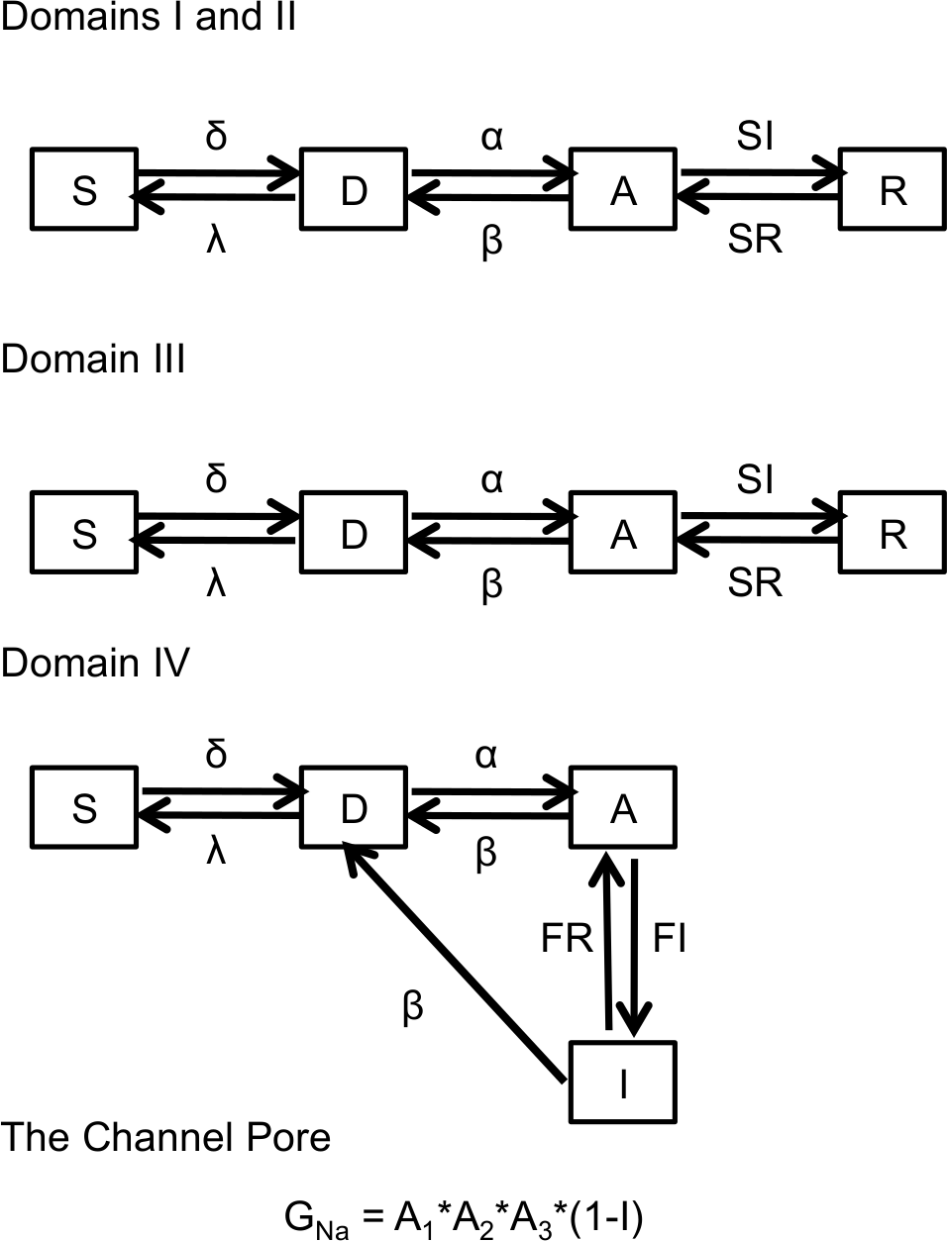
Proposed model of Na_V_1.5 channels. Modelling scheme used to simulate C373F and C373F/E1784K channel ionic and gating currents.

To test whether changes to fast inactivation were sufficient to cause the conductance curve to shift in the depolarizing direction, we simulated our sodium currents using a novel tetrameric model of the sodium channel voltage sensors linked by a Hodgkin and Huxley-style equation to model the pore (Fig 6). Data from our ionic and gating current recordings was used to fit the rates of the 4 individual voltage sensors (Table 1).

**Table 1:** Transition rates for the peters-ruben model.

	CF pH 7.4	CF/EK pH 7.4	CF pH 6.0	CF/EK pH 6.0
**δ**	2.2897	2.2897	1.922	1.922
**λ**	22.897	22.897	19.22	19.22
**α**_**1**_	381.14*e*^*V*/9.657^	381.14*e*^*V*/9.657^	371.54*e*^*V*/7.813^	371.54*e*^*V*/7.813^
**β**_**1**_	0.3446*e*^*V*/−78.1^	0.3446*e*^*V*/−78.1^	0.104*e*^*V*/−49.96^	0.104*e*^*V*/−49.96^
**α**_**2**_	381.14*e*^*V*/9.657^	381.14*e*^*V*/9.657^	371.54*e*^*V*/7.813^	371.54*e*^*V*/7.813^
**β**_**2**_	0.3446*e*^*V*/−78.1^	0.3446*e*^*V*/−78.1^	0.104*e*^*V*/−49.96^	0.104*e*^*V*/−49.96^
**α**_**3**_	183.94*e*^*V*/22.4^	183.94*e*^*V*/22.4^	134.71*e*^*V*/10.73^	134.71*e*^*V*/10.73^
**β**_**3**_	0.3867*e*^*V*/−38.76^ × (*DIIIA* – *DIVI*)	0.3867*e*^*V*/−38.76^ × (*DIIIA* – *DIVI*)	0.1025*e*^*V*/−30.29^ × (*DIIIA* – *DIVI*)	0.1025*e*^*V*/−30.29^ × (*DIIIA* – *DIVI*)
**α**_**4**_	91.93*e*^*V*/8.417^ + 48.39*e*^*V*/11.51^	152*e*^*V*/11.8^ + 60.88*e*^*V*/12.97^	237*e*^*V*/7.207^ + 4.99*e*^*V*/16.01^	204.65*e*^*V*/11.02^ + 19.59*e*^*V*/15.31^
**β**_**4**_	9.273 × 10^−6^*e*^*V*/−11.51^	3.904 × 10^−5^*e*^*V*/−12.52^	1.432 × 10^−5^*e*^*V*/−11.51^	1.144 × 10^−4^*e*^*V*/−14^
**I**_**1**_	9.39 × 10^−5^	9.39 × 10^−5^	4.278 × 10^−5^	4.278 × 10^−5^
**R**_**1**_	3.874 × 10^−4^ + 4.687 × 10^−6^*e*^−*V*/15.06^	3.874 × 10^−4^ + 4.687 × 10^−6^*e*^−*V*/15.06^	3.597 × 10^−4^ + 3.666 × 10^−6^*e*^*V*/−14.32^	3.597 × 10^−4^ + 3.666 × 10^−6^*e*^*V*/−14.32^
**I**_**2**_	9.39 × 10^−5^	9.39 × 10^−5^	4.278 × 10^−5^	4.278 × 10^−5^
**R**_**2**_	3.874 × 10^−4^ + 4.687 × 10^−6^*e*^−*V*/15.06^	3.874 × 10^−4^ + 4.687 × 10^−6^*e*^−*V*/15.06^	3.597 × 10^−4^ + 3.666 × 10^−6^*e*^*V*/−14.32^	3.597 × 10^−4^ + 3.666 × 10^−6^*e*^*V*/−14.32^
**I**_**3**_	3.971 × 10^−5^	3.971 × 10^−5^	2.716 × 10^−5^	2.716 × 10^−5^
**R**_**3**_	5.199 × 10^−5^ + 2.642 × 10^−6^*e*^*V*/−22.47^	9.436 × 10^−5^ + 2.396 × 10^−6^*e*^*V*/−18.97^	3.852 × 10^−5^ + 9.863 × 10^−7^*e*^*V*/−17.85^	4.994 × 10^−5^ + 6.335 × 10^−7^*e*^*V*/−14.21^
**FI**	1.522	2.50	1.178	1.71
**FR**	0.002	0.024	0.0015	0.023

V is membrane potential in mV and all rates are in ms^-1^

The forward voltage-independent rate between the S and D states is the maximal rate of outward gating charge movement.

The D to A transition rates for the DI-DIII VSD were derived from the time constants of gating charge movement. Initially, we fit the 3 forward and reverse rates with the same equation, which produced recognizable sodium currents. To provide an accurate fit of ionic currents, we used a genetic algorithm followed by a climbing hill algorithm to minimize the deviation between simulated ionic currents and averaged experimental currents. These algorithms were performed with the constraint that the DI and DII VSD are equivalent and therefore their motion can be fit by the same parameters. The outcome of the two algorithms was a shift in the steady-state activation of the DIII VSD to more hyperpolarized potentials.

The D to A transition rates of the DIV VSD were modeled from fast inactivation time constants and steady-state values at given membrane potentials. The voltage-independent inactivation rate between the DIV-A and DIV-I states was the maximum rate of open-state fast inactivation in the experiments. To model charge immobilization, the deactivation rate for DIII VSD was multiplied by one minus the number of DIV VSD in the I state; the result was immobilization of the DIII voltage sensor leading to a slower deactivation rate of DIII when the channel is inactivated.

The relaxation and de-relaxation rates of DIS4 and DIIS4 were directly derived from the fast time constants of slow inactivation, whereas relaxation and de-relaxation rates of DIIIS4 were derived from the slow component of slow inactivation [8,9]. The relaxation rates at -30 mV, 0 mV, and 30 mV were relatively similar, thus we used the average of the three as a voltage-independent forward rate. The relaxation and de-relaxation rates of Domains I and II were multiplied by 0.707 (the square root of 0.5) to reflect that relaxation of either of the two VSD was sufficient to cause slow inactivation[8,9].

### The model simulates both ionic and gating currents

**Fig 7:**
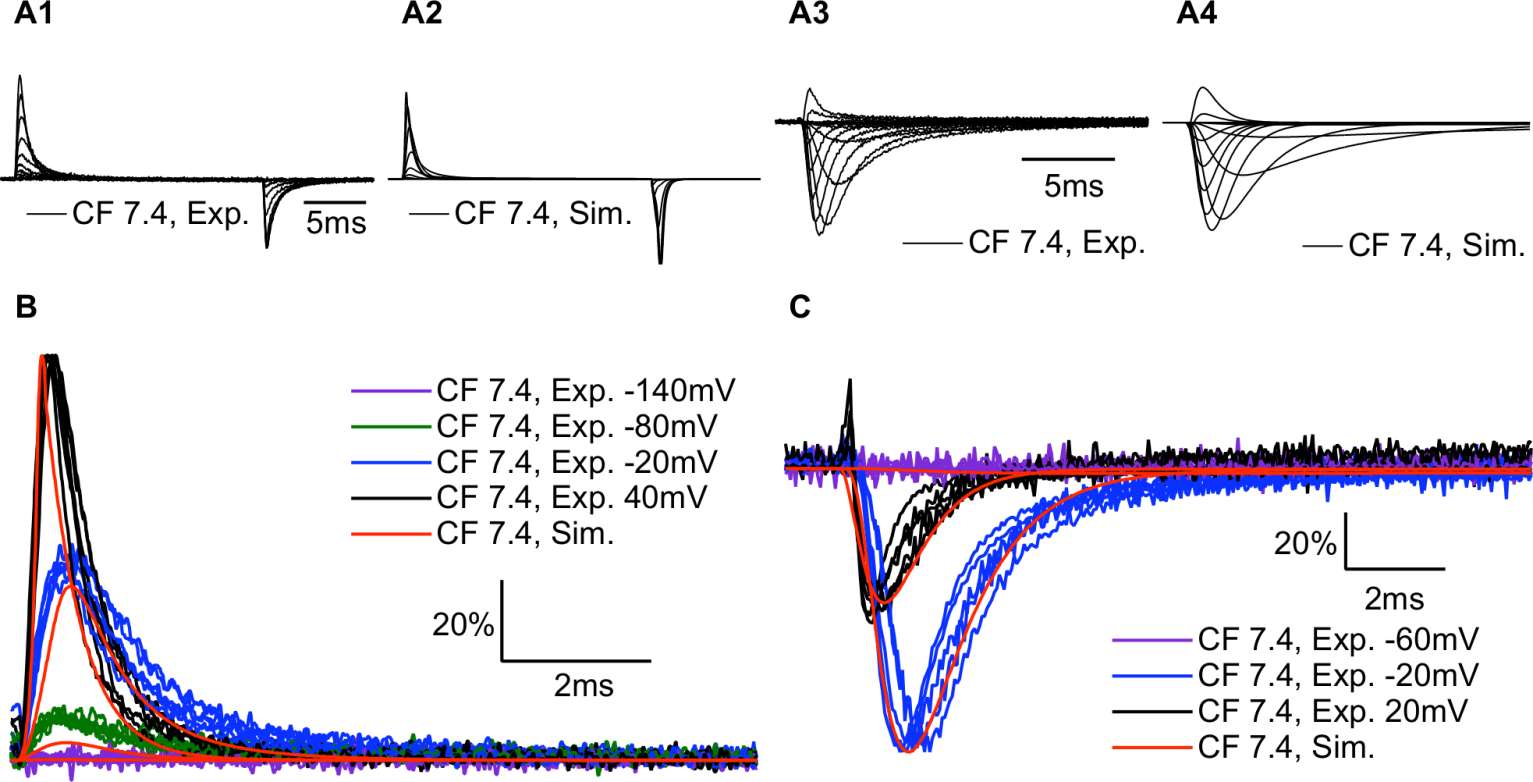
Modeled gating currents and ionic currents are similar to experimental recordings. **(A1-4)** Sample experimental and simulated gating currents and ionic currents for C373F Na_V_1.5 at pH 7.4. **(B)** Overlaps of outward gating current traces recorded from 6 different cells expressing C373F Na_V_1.5 at pH 7.4 with simulated outward gating currents at the same membrane potentials. Gating currents were normalized to the peak outward current at 40mV. **(C)** Overlaps of ionic current traces recorded from 6 different cells expressing C373F Na_V_1.5 at pH 7.4 with simulated ionic currents at the same membrane potentials. Ionic currents were normalized to the peak inward current at 20mV. Overall the model replicates the experimental ionic and gating currents at different membrane potentials.

Experimental and simulated gating currents (Fig 7A1 and 7A2) and ionic currents (Fig 7A3 and 7A4) are shown for C373F channels at pH 7.4. To produce modeled gating current traces, we calculated the proportion of each voltage sensor which shifted between the D and A states within a given time step. We multiplied the values for DIII and DIV by 1.5 to account for the 50% larger gating charge moved by these two voltage sensors and the resultant values were summed with those from DI and DII [47]. Our model accurately reproduces the time course of our experimental gating currents (Fig 7B) and ionic currents (Fig 7C).

### The model accurately simulates activation and fast inactivation at pH 7.4 and pH 6.0

**Fig 8:**
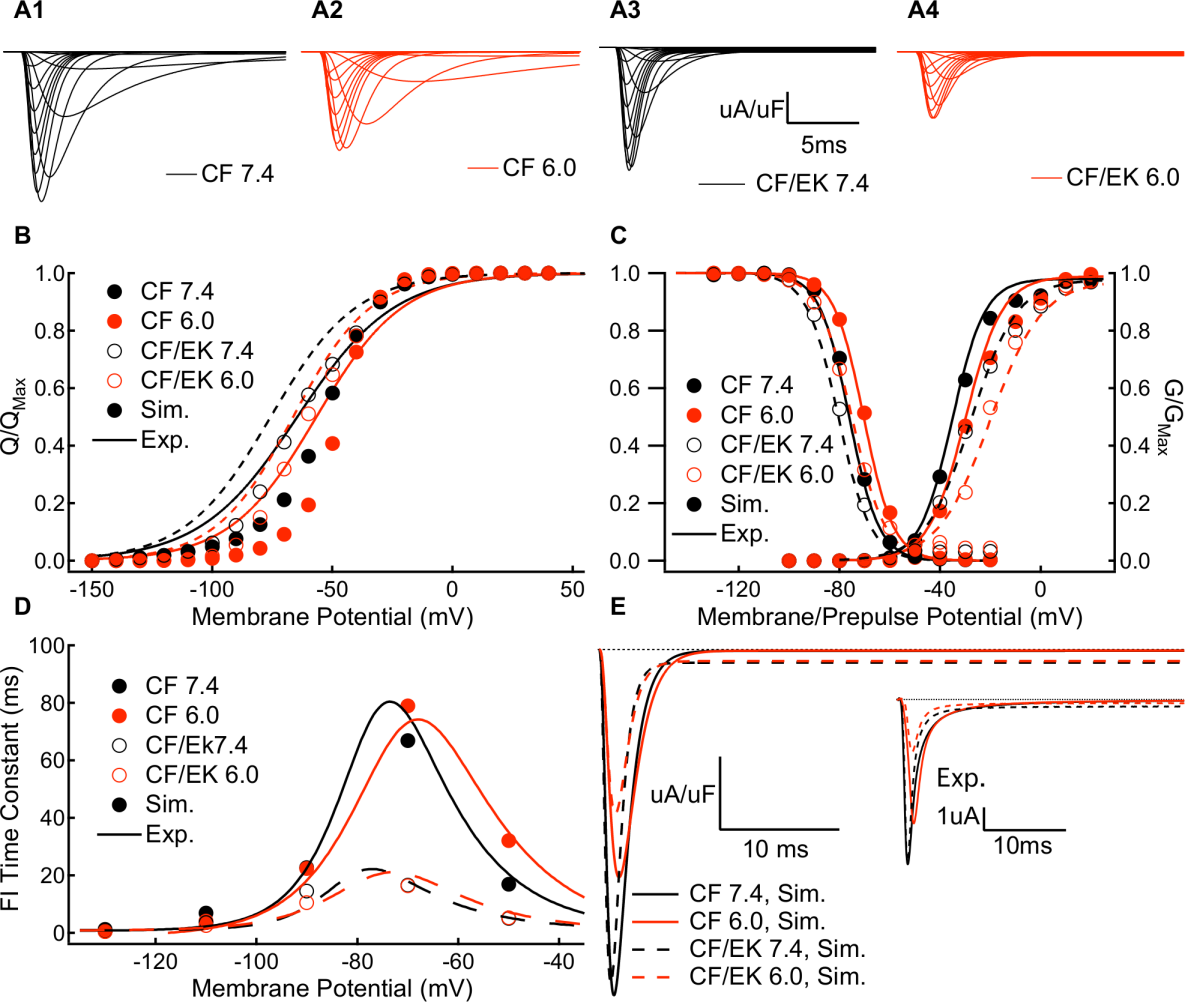
Modeled C373F and C373F/E1784K channels show similar gating parameters and persistent currents compared to experiments. **(A1-A4)** Simulated ionic currents from C373F and C373F/E1784K Na_V_1.5 models at pH 7.4 and pH 6.0. **(B)** Simulated outward charge-voltage relationships from C373F (solid circles) and C373F/E1784K (open circles) Na_V_1.5 models at pH 7.4 (black) and pH 6.0 (red) are overlapped with fits to data from C373F (solid lines) and C373F/E1784K (dashed lines) experiments at pH 7.4 (black) and pH 6.0 (red). In all cases, there is a small depolarizing shift in the simulated gating current voltage-dependence which is reviewed in the discussion section **(C)** Simulated fast inactivation steady-state and conductance-voltage relationships from C373F (solid circles) and C373F/E1784K (open circles) Na_V_1.5 models at pH 7.4 (black) and pH 6.0 (red) overlapped with fits to data from C373F (solid lines) and C373F/E1784K (dashed lines) experiments at pH 7.4 (black) and pH 6.0 (red). **(D)** Simulated time constants of fast inactivation recovery (-130mV to -70mV) and closed-state fast inactivation onset (-70mV and -50mV) from C373F (solid circles) and C373F/E1784K (open circles) Na_V_1.5 models at pH 7.4 (black) and pH 6.0 (red) overlapped with fits to data from C373F (solid lines) and C373F/E1784K (dashed lines) experiments at pH 7.4 (black) and pH 6.0 (red). **(E)** Simulated persistent currents at -20mV from C373F and C373F/E1784K Na_V_1.5 models at pH 7.4 and pH 6.0. (**E *inset***) Sample experimental persistent current recordings at -20mV from C373F and C373F/E1784K Na_V_1.5 at pH 7.4 and pH 6.0. Overall the model accurately replicates the experimental conductance-voltage relationship, steady-state fast inactivation, time course of fast inactivation, and persistent current amplitudes.

We used the modeling scheme in Fig 6 and the same approach discussed above to model CF channels at pH 6.0. To model CF/EK channels at pH 7.4 and pH 6.0, we modified only the rates of DIV VSD movement from the CF model at the same pH to correspond to the experimental fast inactivation data from CF/EK channels. The E1784K model also includes a modified de-relaxation rate of DIII, but not domains I or II, to reflect the changes to the slow time component of slow inactivation, although this was not necessary to model channel conductance or fast inactivation. Simulated macroscopic currents from CF and CF/EK models at pH 7.4 and pH 6.0 are shown (Fig 8A1–8A4).

By integrating the modeled gating current, we obtained modeled charge-voltage (QV) curves (Fig 8B). All 4 models show a consistent, small shift of the QV relationship in the depolarizing direction which is discussed in the model limitations section of the discussion.

The model accurately replicates experimental conductance-voltage relationships (Fig 8C), fast inactivation voltage-dependence (Fig 8C), fast inactivation time constants (Fig 8D), and persistent currents (Fig 8E).

The model also simulates multi-time component slow inactivation recovery and onset (S4 Fig).

### The E1784K mutant alters channel conductance by modifying fast inactivation

**Fig 9:**
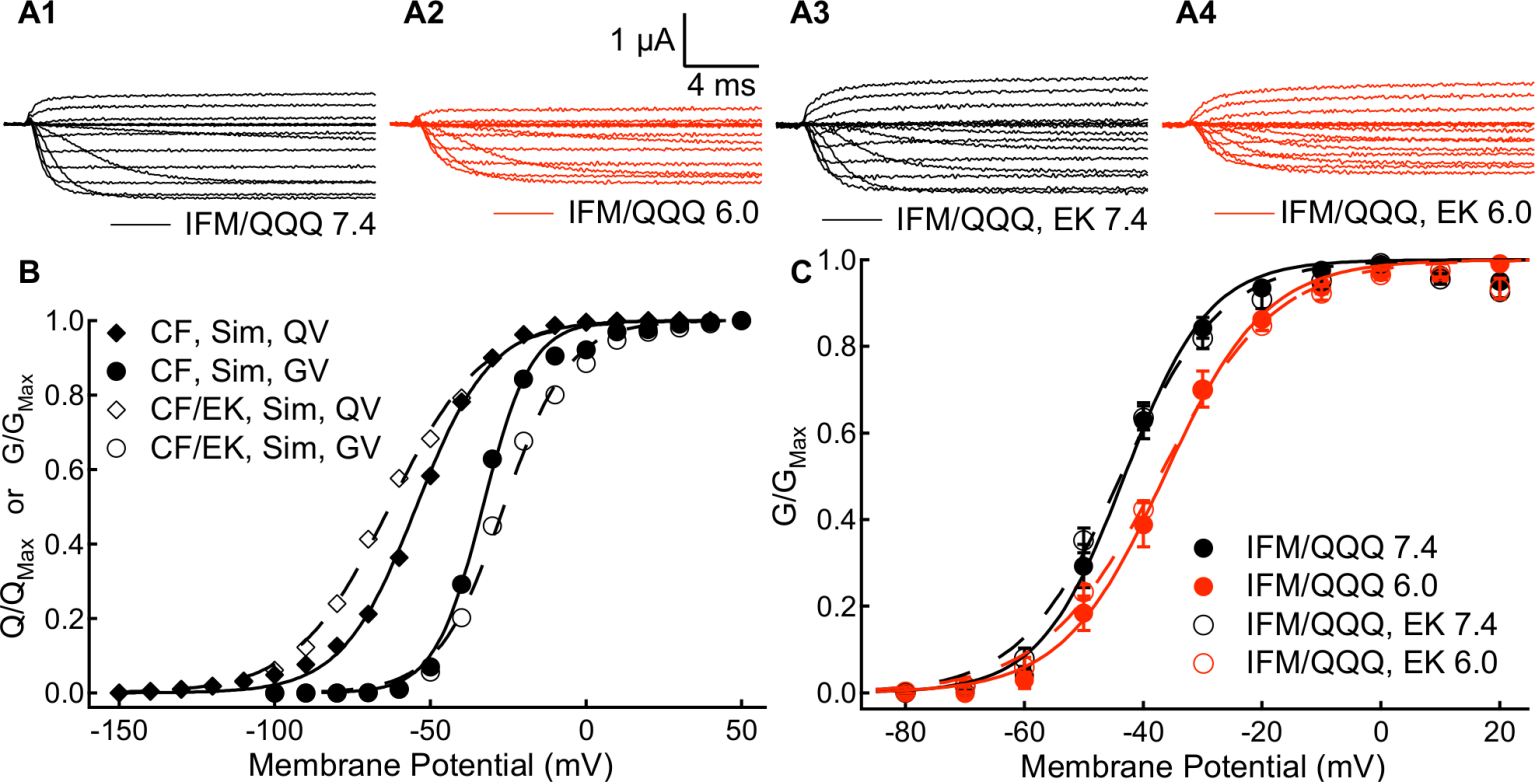
E1784K induced changes to channel fast inactivation are sufficient to depolarize the conductance-voltage relationship. **(A1-A4)** Sample ionic currents recorded from IFM/QQQ and IFM/QQQ-E1784K Na_V_1.5 at pH 7.4 and pH 6.0. **(B)** Simulated outward charge-voltage and conductance-voltage relationships from C373F and C373F/E1784K Na_V_1.5 models at pH 7.4. To replicate the hyperpolarizing shift in the midpoint of the charge-voltage relationship and the depolarizing shift in the midpoint of the conductance voltage-relationship in the E1784K model required only changes to DIVS4 movement to correspond to experimental data on fast inactivation **(C)** Conductance-voltage relationships of IFM/QQQ and IFM/QQQ-E1784K Na_V_1.5 at pH 7.4 and pH 6.0. In the absence of fast inactivation, the E1784K mutant does not significantly affect the conductance-voltage relationship.

As hypothesized, our model reproduced the depolarizing shift in the midpoint of the C373F/E1784K conductance curve without any modifications in the activation rates of DI-DIII (Fig 9B). This mutant-dependent depolarizing shift was accompanied by a shift in the QV curve in the E1784K model to more hyperpolarized membrane potentials, consistent with the shift seen experimentally. To confirm that the changes to channel conductance in the E1784K mutant are due to altered fast inactivation, we recorded currents in the fast inactivation-deficient IFM/QQQ and the IFM/QQQ/E1784K mutants (Fig 9A1–9A4). In the absence of fast inactivation, the conductance voltage relationship in the IFM/QQQ/E1784K mutant are not significantly different from those of IFM/QQQ (Fig 9C and Table J in the S1 Appendix). These results confirm the prediction of our simulations, specifically that E1784K modifies channel conductance through changes to the DIV-VSD which shift the voltage dependence of fast inactivation to more hyperpolarized potentials and accelerate the rate of fast inactivation.

## Discussion

### Acidosis provides a further arrhythmogenic substrate in E1784K carriers

Although congenital LQT3 and BrS1 patients carry SCN5A mutations from birth, their hearts may beat billions of times without an arrhythmic event; yet when it does occur a single event is sufficient to cause sudden cardiac death. This event may be triggered by physiological changes that exacerbate the effects of these mutants, causing the electrical instability necessary for an arrhythmia to occur.

Acidaemia is a common factor in a number of scenarios which can elicit arrhythmia and sudden cardiac death, including sudden infant death syndrome, exercise, cocaine ingestion, and ischemic heart disease [16,29–33]. During myocardial ischemia, extracellular pH may drop as low as pH 6.0, and exercise can produce decreases to pH 7.0–7.2 [36,48,49]. Cocaine usage may cause acute pH as low as 6.5 and can elicit a Brugada-like ECG in the absence of an underlying SCN5A mutation, a condition termed Brugada-phenocopy [29,34,50]. Lowered extracellular pH decreases peak sodium currents and increases the fraction of non-inactivating channels, similar to Na_V_1.5 mutants which cause BrS1 and LQT3, respectively [27]. Thus, acidaemia may act as a trigger for arrhythmia in patients who already carry BrS1 or LQT3 mutant channels.

A previous study from our lab identified the most common LQT3/BrS1 mixed mutant, E1784K, as being preferentially sensitive to changes in extracellular pH [16,22]. Consistent with our previous study, we show that lowered extracellular pH produces a larger shift of the conductance curve in the depolarizing direction and a larger proton dependent block of inward sodium currents in E1784K compared to WT Na_V_1.5 [16]. The depolarizing shift in the midpoint of conductance when extracellular pH was decreased from pH 7.4 to pH 6.0 is twice as large (≈8.5 mV) in the CF/EK Na_V_1.5 compared to CF Na_V_1.5 (≈4 mV). The larger depolarizing shift seems to be limited to low extracellular pH below pH 7.0 as both CF and CF/EK channels had similar (≈1.8 mV) shifts when pH was decreased to pH 7.0. The effect was similar with respect to proton dependent decreases in channel conductance as CF and CF/EK Na_V_1.5 show relatively similar effects at pH 7.0, 3% block in CF and 1% in CF/EK, when compared to the block at pH 6.0, 26% in CF and 37% in CF/EK. Overall these results suggest that, as pH decreases below pH 7.0, the CF/EK mutant will show significantly larger decreases in inward sodium current compared to CF Na_V_1.5. In patients, acidaemia could exacerbate the decreases in peak current found with the E1784K mutant, potentially increasing the risk of arrhythmia or sudden cardiac death.

### The E1784K mutant does not alter channel conductance through changes to voltage-sensor activation

As low extracellular pH is known to modulate the movement of the channel voltage-sensors [51], we measured gating currents to test whether altered voltage-sensor movements underlie the mutant- and pH-dependent affects of the E1784K mutant. We hypothesized that the depolarizing shift of the conductance curve in the E1784K mutant is caused by a depolarizing shift in the voltage at which S4s translocate. Our gating current experiments show that this hypothesis is unlikely to be true, since the QVon relationship is shifted in the hyperpolarizing direction in the CF/EK mutant compared to CF. We therefore conclude that the shifts in the channel conductance curve in the E1784K mutant are not due to a depolarizing shift of the movements of the DI-DIII S4s, which are linked to channel activation [52].

### The E1784K mutant alters channel fast inactivation

Recently, the first crystal structure of a eukaryotic voltage-gated sodium channel showed coupling between the channel C-terminus, the DIII-DIV linker, and the cytoplasmic regions of DIV [53]. This supports the evidence from previous studies that showed the C-terminus plays an important role in regulating channel fast inactivation [54,55]. To test whether the effects of the E1784K mutant on conductance and gating currents are consistent with changes to fast inactivation, we measured voltage-sensor fluorescence and created a new model of voltage-gated sodium channel behavior that can simulate both ionic and gating currents. Voltage-clamp fluorimetry reveals that the movement of DIVS4 is shifted in the hyperpolarizing direction by the E1784K mutant, which is consistent with the hyperpolarizing shift of the steady-state fast inactivation relationship and the hyperpolarizing shift in the movement of gating charge. We used our model to test whether modifications of DIVS4 were sufficient to explain a hyperpolarizing shift of the charge-voltage relationship and the depolarizing shift of the conductance-voltage relationship. The increased probability and rate of FI in the mutant could lead to decreased peak sodium currents in the middle of the normal conductance curve when the rates of opening are slowest. This hypothesis is also consistent with data that shows a significant number of channels enter the fast inactivated state before channel activation is complete [56].

When we altered the DIV VSD rates to correspond to the increased probability and rate of FI in E1784K, the simulated GV curve shifted to more depolarized potentials to accurately reproduce the experimental GV. Furthermore, when we removed channel fast inactivation experimentally with IFM/QQQ, the E1784K mutant no longer shifted the GV curve. Taken together, these data suggest that changes to channel fast-inactivation are sufficient to explain E1784K effects on channel conductance.

Localizing E1784K effects to fast inactivation may be important for targeting therapeutics to patients. As with some other Na_V_1.5 mutants, E1784K is associated with both a gain-of-function disorder (LQT3) and a loss-of-function disorder (BrS1) [22,57]; pharmacological interventions for these mixed syndrome mutants may prove difficult [58]. One of the first LQT3-specific treatments was the sodium channel blocker mexiletine; however, not all LQT3 patients respond to mexiletine, and its effects in BrS1 patients are mixed [59]. Mexiletine rescues some traffic deficient mutants, thereby increasing current density. However, in one patient with both BrS1 and LQT3 mexilitine was found to increase persistent currents while also blocking the peak currents in the channel, exacerbating properties associated with BrS1 and leading to cardiac arrhythmia [60]. These cases illustrate that, although the SCN5A gene is a common factor for BrS1 and LQT3 patients, the pathophysiology and response to pharmaceuticals can vary based on the specific mutant. We suggest that E1784K alters fast inactivation, thus pharmaceuticals targeted at DIV are a rational direction for drug development in E1784K patients.

### The E1784K mutant destabilizes charge immobilization

Interestingly, we found that despite the hyperpolarization of gating current activation in CF/EK channels, the voltage-dependence of gating current deactivation is shifted in the depolarizing direction in E1784K. The result is that the large hysteresis between voltage-sensor activation and deactivation observed in CF channels (≈20mV) is not apparent in CF/EK channels (≈5mV). This may reflect the decreased affinity of the IFM fast inactivation particle and, therefore, a decreased fraction of charge which is normally immobilized by channel fast inactivation [44,61]. This idea is consistent with the decreased amplitude of the slow component of gating charge deactivation we observed in CF/EK channels. Overall, these results, combined with the increased rate of fast inactivation recovery, suggest a lower energy barrier for the deactivation of the DIV VSD, which may explain the increases in persistent current in the E1784K mutant.

Similar to other studies, we show that E1784K increases persistent current [16,22,23]. Relative persistent current is further increased at pH 6.0 in the CF/EK channels, while it is relatively similar in CF Na_V_1.5 at pH 7.4 and pH 6.0. Although lowered extracellular pH decreases the absolute peak and persistent current amplitudes, persistent current is more resistant to pH changes than peak current, hence the increase in relative persistent current. Thus, although we predict that protons will block sodium channels and increase the likelihood of conduction abnormalities in patients carrying the E1784K mutant, this blockade may not sufficiently reduce persistent currents to alleviate the elongated AP plateau.

The increased persistent current in E1784K suggests that channels can more readily reopen from the fast-inactivated state in E1784K. This decreased stability of the fast-inactivated state may reflect a less stable binding by the IFM motif or an accelerated fast inactivation recovery rate in E1784K. Interestingly, the increase in persistent current occurs in the presence of a hyperpolarizing shift of the fast inactivation voltage-dependence, which normally indicates a stabilization of fast inactivation; however, a hyperpolarizing shift of the fast inactivation voltage-dependence does not actually correlate well with a decrease in persistent current. Many other C-terminal mutants, including V1777M, 1795insD, and L1825P, show similar hyperpolarizing shifts with an increase in persistent current [62–64]. Conversely, in some mutants, calcium shifts the steady-state fast inactivation curve in the depolarizing direction and decreases persistent sodium currents [65,66]. This may reflect the fact that fast inactivation occurs in two steps. Voltage-dependence is conferred by the DIVS4 voltage-sensors which, when active, allows binding of the IFM motif to cause fast inactivation [6,45]. Therefore, stabilization of the DIVS4 in the activated state could theoretically occur with less stable binding of IFM and, consequently, an increase in persistent current. This less stable binding could be due to changes to the binding site when the DIVS4 is altered, or due to rapid recovery of the DIVS4.

### The E1784K mutant accelerates slow inactivation

We show that the E1784K mutant accelerates onset and recovery of a component of slow inactivation in the 1-10s range. We speculate that, because the E1784K channels show both accelerated onset and recovery from slow inactivation, there will be little effect on channel availability at resting heart rates. Although a greater number of channels will enter the slow inactivated state during the action potential plateau, the long diastolic time and accelerated recovery rate will be sufficient to recover this extra slow inactivation; however, as heart rate increases and the diastolic interval represents a smaller proportion of each cycle, the increased slow inactivation in E1784K may not fully recover, which would decrease the number of available sodium channels. This could decrease peak sodium currents, slow conduction velocity, and increase the propensity of E1784K to cause a Brugada syndrome waveform.

### The Peters-Ruben model

We chose to construct a novel tetrameric model of sodium channel gating which uses 4, 4-state chains to model the individual voltage-sensors. Similar to hERG potassium channel models, our model includes a voltage-independent step in activation [67]. This step constrains the activation rates at depolarized potentials as channel activation rates plateau at non-zero values (S2 Fig) [51,52]. Our model also includes a relaxed state, simulating slow inactivation. Fluorescence and gating current measurements show that the relaxed state is present in sodium and potassium channels, and, given its presence in *Ciona intestinalis* voltage-sensing phosphatase, is likely an intrinsic voltage-sensor property [8,9,68–70]. In sodium channels, voltage-sensor relaxation is correlated to slow inactivation of ionic currents [8,9].

Tetrameric models of channel gating which focus on modeling voltage-sensor states have advantages and disadvantages when compared to Markov models of channel states. Tetrameric models of the voltage sensor states are more efficient than Markov models of the entire channel [71]. The expanded Markov model of our 16-state scheme would require 256 states (4^4^). Previous authors have reduced the number of states in Markov models by omitting slow inactivation [52,72]. Another method to reduce the number of states is to assume that all voltage sensors are equivalent to one another [73]; this assumption, however, is in disagreement with structural and fluorescence data [3,8,52,53]. Using a tetrameric model allowed us to model non-equivalent voltage-sensors with relaxation transitions that can describe multi-time component slow inactivation, as has been shown experimentally [8,9].

A disadvantage of tetrameric models is some difficulty in modeling interaction effects and concerted movements. This situation has been studied previously in terms of concerted pore opening of potassium channels, with the authors proposing an efficient algorithm to exactly model the results from a Markov model with a tetrameric model [71]. An important interaction in sodium channels is the immobilization of the DIIIS4 and DIVS4 by fast inactivation [44]. Recent fluorescence data shows that during longer depolarizations the deactivation rate of DIIIS4 is strongly correlated to the rate of recovery from fast inactivation [74]. Whether DIIIS4 deactivation is responsible for fast inactivation recovery or fast inactivation recovery driven by DIVS4 deactivation allows for DIIIS4 deactivation is still unresolved and may differ between channel isoforms [45,74]. In our model, this interaction was modeled by making the deactivation rate in DIII inversely proportional to the fraction of DIV sensors in the inactivated state. We did not explicitly include other cooperativity between voltage-sensors as we assume that the coupling between sensors is implicit in the experimental data from which our model is derived [46].

Overall, our model simulates realistic gating and ionic currents including slow inactivation. This is in contrast to many other models which are not mechanistically parameterized with gating current data, or do not include any data related to deeper inactivated states [52,73,75,76].

One limitation of our model is a consistent depolarizing shift of the charge-voltage relationship in all conditions. This may be due to us not assigning the correct relative amounts of gating charge to each voltage-sensor. Based on estimates of total charge contribution from experiments using lidocaine to trap voltage-sensors, we assigned 30% of the total charge moved to each of DIIIS4 and DIVS4 and 20% to each of DIS4 and DIIS4 [47]. This assumption may be incorrect, however, as fluorescence experiments suggest DIIS4 and DIIIS4 may be the voltage-sensors which carry the most charge [3]. It is also possible that constraining DIS4 and DIIS4 to be equivalent causes part of the depolarizing shift. DIS4 movement is hyperpolarized compared to DIIS4, although not to the extent of DIIIS4, and this would also shift the QV to more hyperpolarized potentials [3].

## Conclusion

Overall, our results (1) show profound effects of decreasing extracellular pH on a relatively prevalent SCN5A mutation, and (2) suggest that the mechanism by which E1784K alters channel function is tied primarily to the fast inactivation mechanism. This second finding is consistent with the location of the mutant in the C-terminus, a region shown to interact with the DIII-DIV linker and to play a key role in determining the rates of fast inactivation [6,53,55,65]. Understanding the mechanism by which E1784K alters channel gating may also act as a general indicator for how C-terminal sodium channel mutants lead to disease. Other mutants in this region cause similar changes in persistent current, channel activation, and the voltage-dependence of fast inactivation. In particular the 1795insD founder mutant is also linked to both Brugada syndrome and long QT syndrome 3 and causes a similar depolarizing shift of the channel conductance curve, hyperpolarizing shift e of the channel inactivation voltage-dependence, and increase in persistent current [57,62]. Understanding the pathophysiology of the C-terminal mutants may allow for more specific drug targeting. Drugs that can restore the movement of DIVS4 may be capable of treating both the increases in persistent current and decreases in peak current in these mutants.

## Supporting information

S1 Appendix Source data for figures. Table A. Source data for the voltage-dependence of channel conductance and fast inactivation. Table B. Source data for fast inactivation recovery and closed-state inactivation onset time constants. Table C. Source data for open state fast inactivation time constants. Table D. Source data for persistent sodium currents. Table E. Source data for slow inactivation recovery time constants of C373F and C373F/E1784K channels. Table F. Source data for slow inactivation onset time constants of C373F and C373F/E1784K channels. Table G. Source data for gating charge activation and deactivation voltage-dependence. Table H. Source data for gating current deactivation recovery. Table I. Source data for fluorescence voltage-dependence of DIII and DIV VCF constructs. Table J. Source data for IFM/QQQ and IFM/QQQ-EK conductance voltage-dependence.
(PDF)

S1 Fig Intracellular pH changes during extracellular acidosis. Intracellular pH was measured for 5 minutes with extracellular pH at pH 7.4 and for 5 minute after changing extracellular pH to pH 6.0 in (**A**) un-injected cells (N = 5) and cells injected with (**B**) C373F (N = 5) or (**C**) C373F/E1784K (N = 5) Na_V_1.5. Cells were held at -110 mV and were depolarized to 0 mV 60 times during each 5-minute segment. All error bars are standard error of the mean.
(TIFF)

S2 Fig The E1784K mutant does not alter the rate of outward gating charge movement. The rate of outward gating charge determined by fitting the decay of outward gating currents with a single exponential is shown for CF and CF/EK channels at pH 7.4 and pH 6.0.
(TIFF)

S3 Fig Conductance-voltage and fast inactivation voltage-dependence relationships for VCF constructs. Conductance-voltage relationships for DIII (**A**) and DIV (**B**) VCF constructs with and without the E1784K mutant. In both VCF constructs the E1784K mutant depolarizes the conductance-voltage relationship. Steady-state fast inactivation voltage-dependence for DIII (**C**) and DIV (**D**) VCF constructs with and without the E1784K mutant. In both VCF constructs the E1784K mutant causes a hyperpolarizing shift in the steady-state fast inactivation voltage-dependence.
(TIFF)

S4 Fig Simulated and experimental slow inactivation recovery and onset rates of C373F Na_V_1.5. Fits to the simulated slow inactivation recovery time courses at -80 mV after depolarizations to 0 mV ranging between 500 ms (top trace) to 64 s (bottom trace) are overlapped to experimental data (**C**). Fits to simulated slow inactivation onset time courses at 0 mV with a recovery pulse between 100 ms and 10 s to -80 mV are overlapped with experimental data (**D**).
(TIFF)
