# Supplementary material for: Depolarization of the conductance-voltage relationship in the NaV1.5 mutant, E1784K, is due to altered fast inactivation
Source: PLoS One. 2017 Sep 12;12(9):e0184605. doi: 10.1371/journal.pone.0184605 (PMC5595308; doi:10.1371/journal.pone.0184605)
Supplement: S1 Appendix — Table A. Source data for the voltage-dependence of channel conductance and fast inactivation. Table B. Source data for fast inactivation recovery and closed-state inactivation onset time constants. Table C. Source data for open state fast inactivation time constants. Table D. Source data for persistent sodium currents. Table E. Source data for slow inactivation recovery time constants of C373F and C373F/E1784K channels. Table F. Source data for slow inactivation onset time constants of C373F and C373F/E1784K channels. Table G. Source data for gating charge activation and deactivation voltage-dependence. Table H. Source data for gating current deactivation recovery. Table I. Source data for fluorescence voltage-dependence of DIII and DIV VCF constructs. Table J. Source data for IFM/QQQ and IFM/QQQ-EK conductance voltage-dependence. (PDF) [file pone.0184605.s001.pdf]

S1 Appendix: Source Data

Table A. Voltage Dependence of Channel Conductance and Fast Inactivation

	**G $V_{1/2}$ (mV)**	**G z**	**FI $V_{1/2}$ (mV)**	**FI z**
**CF, pH 7.4**	-35.2±1.0	4.14±0.23	-75.4±1.05	-4.10±0.34
**CF, pH 7.0**	-33.5±1.1	4.12±0.20	-73.3±0.7	-4.45±0.25
**CF, pH 6.0**	-29.7±1.1	3.60±0.23	-70.4±0.6	-4.38±0.21
**CF/EK, pH 7.4**	-27.8±1.5	2.94±0.08	-80.4±0.6	-4.16±0.31
**CF/EK, pH 7.0**	-26.0±1.3	3.21±0.10	-77.8±0.8	-4.31±0.28
**CF/EK, pH 6.0**	-19.3±1.3	2.47±0.24	-75.1±1.1	-3.89±0.31

Where values are mean ± standard error of the mean

Where N = 8 for CF and CF/EK GVs and N = 5 and N = 6 for CF and CF/EK FI, respectively

G $V_{1/2}$: Midpoint of the conductance-voltage relationship

G z: Apparent valence of the conductance-voltage relationship

FI $V_{1/2}$: Midpoint of the steady-state fast inactivation relationship

FI z: Apparent valence of the steady-state fast inactivation relationship

Table B. Fast Inactivation Recovery and Closed-State Onset Time Constants

	τ_{Frec} -130 mv (ms)	τ_{Frec} -110 mv (ms)	τ_{Frec} -90 mv (ms)	τ_{Frec} -70 mv (ms)	τ_{FI} -70 mv (ms)	τ_{FI} -50 mv (ms)
**CF, pH 7.4**	2.98±0.17	7.52±0.70	21.6±2.7	81.5±4.4	69.2±6.4	26.5±5.6
**CF, pH 7.0**	3.08±0.15	7.54±0.69	20.7±1.6	79.9±4.9	72.8±7.3	25.9±6.1
**CF, pH 6.0**	2.77±0.32	6.15±0.48	17.4±2.5	65.2±4.7	75.2±9.2	50.6±4.8
**CF/EK, pH 7.4**	1.69±0.08	3.61±0.17	8.7±0.4	21.1±2.6	14.9±1.6	6.8±1.1
**CF/EK, pH 7.0**	1.69±0.04	3.76±0.16	9.0±0.4	22.0±1.8	16.3±1.4	6.9±1.0
**CF/EK, pH 6.0**	1.59±0.09	3.36±0.22	7.9±0.4	23.7±2.7	17.0±1.6	9.3±1.1

Where values are mean ± standard error of the mean

N = 6 for all cases

τ_{Frec}: Time constant of recovery from fast inactivation at a given voltage

τ_{FI}: Time constant of closed-state fast inactivation at a given voltage

Table C. Open State Fast Inactivation Time Constants

	τ_{FI} -30 mV (ms)	τ_{FI} -20 mV (ms)	τ_{FI} -10 mV (ms)	τ_{FI} 0 mV (ms)	τ_{FI} 10 mV (ms)	τ_{FI} 20 mV (ms)	τ_{FI} 30 mV (ms)
**CF, pH 7.4**	1.66±0.07	1.24±0.09	1.03±0.07	0.89±0.07	0.85±0.08	0.83±0.09	0.89±0.09
**CF, pH 7.0**	2.03±0.11	1.36±0.05	1.12±0.07	0.92±0.07	0.86±0.09	0.88±0.09	0.94±0.11
**CF, pH 6.0**	2.30±0.12	1.69±0.06	1.25±0.06	1.03±0.08	0.94±0.10	0.98±0.11	1.00±0.14
**CF/EK, pH 7.4**	1.12±0.09	0.90±0.06	0.79±0.04	0.70±0.04	0.64±0.04	0.63±0.04	0.63±0.05
**CF/EK, pH 7.0**	1.26±0.08	1.02±0.06	0.87±0.04	0.75±0.04	0.68±0.03	0.70±0.04	0.70±0.05
**CF/EK, pH 6.0**	1.47±0.09	1.22±0.06	1.07±0.06	0.94±0.05	0.86±0.07	0.81±0.07	0.80±0.08

Where values are mean ± standard error of the mean

Where N = 7 or 8 for all cases

τ_{FI}: Time constant of open-state fast inactivation at a given voltage

Table D. Persistent Sodium Current

	I_{Nap} -30 mV (%)	I_{Nap} 20 mV (%)	I_{Nap} -10 mV (%)	I_{Nap} 0 mV (%)
**CF, pH 7.4**	0.36±0.02	0.44±0.04	0.40±0.02	0.28±0.03
**CF, pH 7.0**	0.44±0.03	0.49±0.04	0.50±0.06	0.39±0.09
**CF, pH 6.0**	0.37±0.05	0.43±0.05	0.42±0.07	0.28±0.12
**CF/EK, pH 7.4**	4.13±0.43	4.02±0.53	3.67±0.53	3.12±0.48
**CF/EK, pH 7.0**	4.75±0.51	4.50±0.57	4.03±0.60	3.25±0.66
**CF/EK, pH 6.0**	6.36±0.67	6.53±0.60	5.88±0.65	4.85±0.75

Where values are mean ± standard error of the mean

Where N = 6 for CF and N = 8 for EK

I_{Nap}: Persistent sodium current measured as the fraction of current remaining at the end of a 100 ms depolarization to a given membrane potential.

Table E. Slow Inactivation Recovery Time Constants

	CF pH 7.4 (s)	CF pH 7.0 (s)	CF pH 6.0 (s)	CF/EK pH 7.4 (s)	CF/EK pH 7.0 (s)	CF/EK pH 6.0 (s)
**-120 mV τ_f**	0.037±0.009	0.042±0.010	0.090±0.027	0.060±0.007	0.057±0.007	0.046±0.012
**-120 mV τ_s**	1.55±0.37	1.80±0.20	1.27±0.11	0.681±0.057	0.630±0.062	0.361±0.067
**-90 mV τ_f**	0.336±0.074	0.301±0.028	0.699±0.441[a]	0.360±0.050	0.324±0.067	0.282±0.062
**-90 mV τ_s**	5.31±0.77	6.99±0.86	6.29±1.06	2.51±0.43	3.08±1.03	1.32±0.33
**-80 mV τ_f**	0.276±0.023	0.285±0.013	0.250±0.013	0.413±0.034	0.434±0.028	0.551±0.131
**-80 mV τ_s**	4.97±0.44	7.11±0.98	5.76±0.515	3.71±0.17	4.78±0.48	4.21±0.86

All values are represented as mean ± standard error

[a] The mean represented contains a single outlier which did not alter the conclusion from the statistical test, with this point removed the mean and standard error is 0.258 ± 0.032

CF: -120 mV, N = 5; -90 mV, N = 4; -80 mV, N = 5

CF/EK: -120 mV, N = 6; -90 mV, N = 5; -80 mV, N = 5

τ_f: Time constant of the fast component of slow inactivation recovery at a given membrane potential

τ_s: Time constant of the slow component of slow inactivation recovery at a given membrane potential

Table F. Slow Inactivation Onset Time Constants

	CF pH 7.4 (s)	CF pH 7.0 (s)	CF pH 6.0 (s)	CF/EK pH 7.4 (s)	CF/EK pH 7.0 (s)	CF/EK pH 6.0 (s)
**-30 mV τ_f**	1.33±0.39	1.61±0.31	1.98±0.55	1.02±0.31	1.56±0.44	1.80±0.48
**-30 mV τ_s**	10.4±1.24	11.4±1.07	15.4±2.44	6.74±1.14	11.6±2.09	16.2±3.79
**0 mV τ_f**	1.15±0.22	0.975±0.167	2.05±0.40	0.425±0.096	0.518±0.069	0.790±0.199
**0 mV τ_s**	9.96±0.63	11.2±1.78	14.5±1.73	5.32±0.74	6.84±0.67	10.7±1.60
**30 mV τ_s [a]**	6.14±0.28	8.35±0.37	10.5±0.69	5.48±0.55	5.03±0.22	8.10±1.17

All values are represented as mean ± standard error

[a] At +30 mV only a single time constant of slow inactivation could be fit. This time constant corresponded in time scale to the slow components measured at -30 mV and 0 mV.

CF: -30 mV N = 4; 0 mV N = 5; 30 mV N = 5

CF/EK: -30 mV N = 5; 0 mV N = 5; 30 mV N = 6

τ_f: Time constant of the fast component of slow inactivation onset at a given membrane potential

τ_s: Time constant of the slow component of slow inactivation onset at a given membrane potential

Table G. Gating Charge Activation and Deactivation Voltage-Dependence

	QV_{On} $V_{1/2}$ (mV)	QV_{On} z	QV_{Deac} $V_{1/2}$ (mV)	QV_{Deac} z
**CF, pH 7.4**	-64.3±1.5	1.29±0.08	-84.9±1.1	1.73±0.07
**CF, pH 6.0**	-56.5±1.6	1.41±0.09	-77.4±1.6	1.69±0.06
**CF/EK, pH 7.4**	-75.6±1.6	1.44±0.07	-79.8±1.3	1.63±0.05
**CF/EK, pH 6.0**	-66.1±0.8	1.55±0.07	-69.8±1.1	1.69±0.06

Where values are mean ± standard error of the mean

Where N = 6 and N = 10 for CF and CF/EK On QVs, respectively

N = 6 and N = 8 for CF and CF/EK Off QVs, respectively

N = 6 and N = 6 for CF and CF/EK Deactivation, respectively

QV_{On} $V_{1/2}$: Midpoint of the charge-voltage relationship measured using outward gating currents

QV_{On} z: Apparent valence of the charge-voltage relationship measured using outward gating currents

QV_{Deac} $V_{1/2}$: Midpoint of the gating charge deactivation voltage-dependence curve

QV_{Deac} z: Apparent valence of the gating charge deactivation voltage-dependence curve

Table H. Gating Charge Deactivation Rates at -150mV

	τ_F (ms)	A_F	τ_S (ms)	A_S
**CF, pH 7.4**	0.583±0.067	0.604±0.048	3.724±0.357	0.469±0.044
**CF, pH 6.0**	0.538±0.040	0.637±0.053	4.183±0.737	0.463±0.052
**CF/EK, pH 7.4**	0.706±0.064	0.807±0.040	5.741±0.793	0.266±0.041
**CF/EK, pH 6.0**	0.738±0.075	0.834±0.031	4.516±0.686	0.275±0.053

All values are represented as mean ± standard error

Where N = 9 for CF and N = 13 for CF/EK

τ_f: Time constant of the fast component of gating charge deactivation

τ_s: Time constant of the slow component of gating charge deactivation

A_F: Amplitude of the fast component of gating charge deactivation

A_S: Amplitude of the slow component of gating charge deactivation

Table I. Voltage-Dependence of Fluorescence for DIII and DIV VCF C373Y and C373Y/E1784K Channels

	**F $V_{1/2}$ (mV)**	**F z**
**DIII VCF CY**	-127.7±1.3	0.82±0.05
**DIII VCF CY/EK**	-135.3±2.0	1.22±0.09
**DIV VCF CY**	-56.8±6.6	1.91±0.32
**DIV VCF CY/EK**	-85.3±4.6	1.60±0.12

All values are represented as mean ± standard error

Where N = 4 for all cases

F $V_{1/2}$: Midpoint of the fluorescence voltage relationship

F z: Apparent valence of the fluorescence voltage relationship

Table J. Voltage-Dependence of IFM/QQQ and IFM/QQQ-EK Conductance

	**G $V_{1/2}$ (mV)**	**G z**
**IFM/QQQ, pH 7.4**	-43.7±1.4	4.10±0.26
**IFM/QQQ, pH 6.0**	-36.8±1.6	3.33±0.24
**IFM/QQQ-EK, pH 7.4**	-44.6±1.0	3.39±0.22
**IFM/QQQ -EK, pH 6.0**	-37.8±0.7	2.77±0.13

Where values are mean ± standard error of the mean

Where N = 7 for IFM/QQQ and IFM/QQQ-EK

G $V_{1/2}$: Midpoint of the conductance-voltage relationship

G z: Apparent valence of the conductance-voltage relationship
