# Supplementary figures and images for: Depolarization of the conductance-voltage relationship in the NaV1.5 mutant, E1784K, is due to altered fast inactivation
Source: PLoS One. 2017 Sep 12;12(9):e0184605. doi: 10.1371/journal.pone.0184605 (PMC5595308; doi:10.1371/journal.pone.0184605)

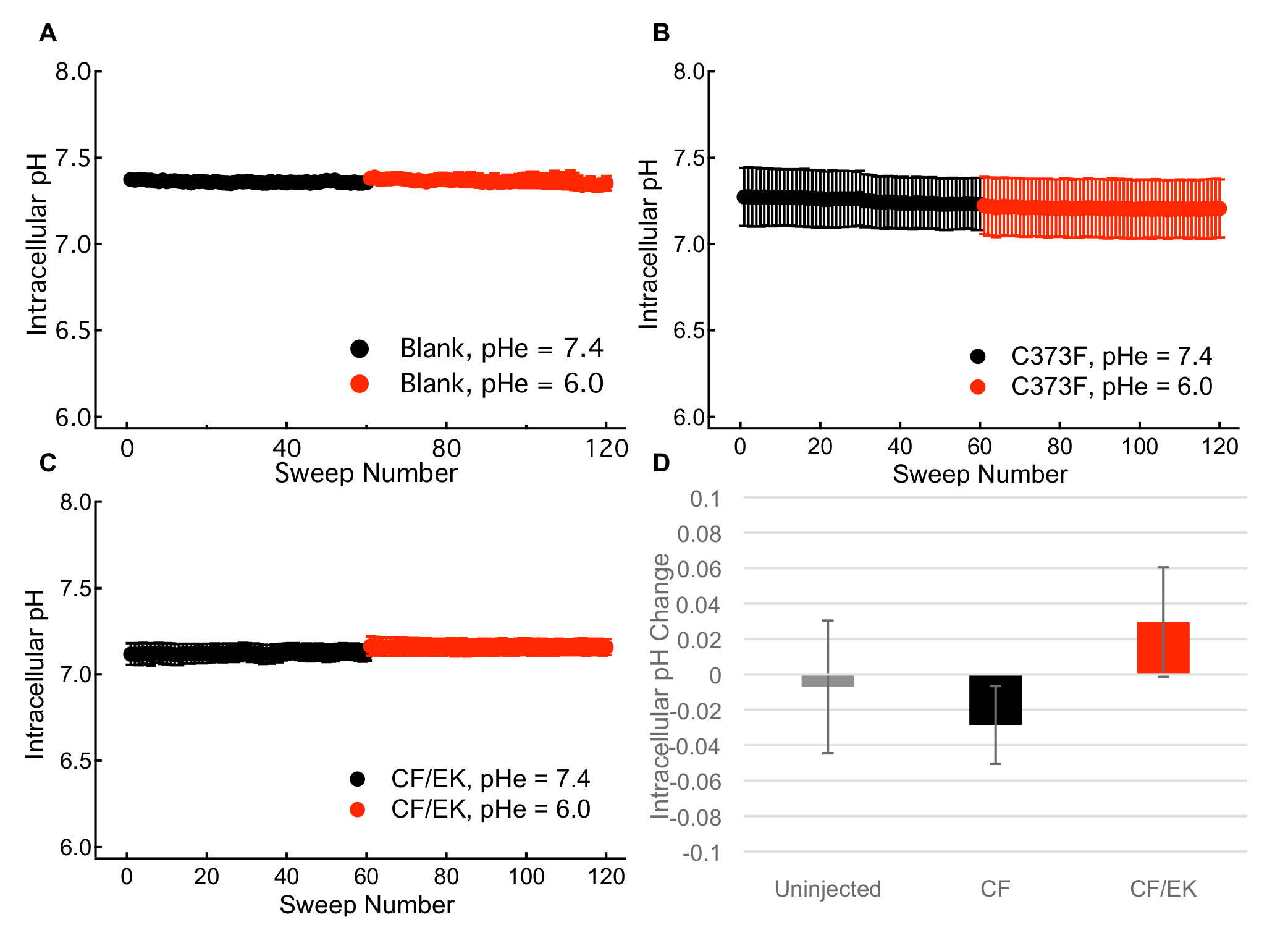

Supplement: S1 Fig — Intracellular pH was measured for 5 minutes with extracellular pH at pH 7.4 and for 5 minute after changing extracellular pH to pH 6.0 in (A) un-injected cells (N = 5) and cells injected with (B) C373F (N = 5) or (C) C373F/E1784K (N = 5) NaV1.5. Cells were held at -110 mV and were depolarized to 0 mV 60 times during each 5-minute segment. All error bars are standard error of the mean. (TIFF) [file pone.0184605.s002.tiff]

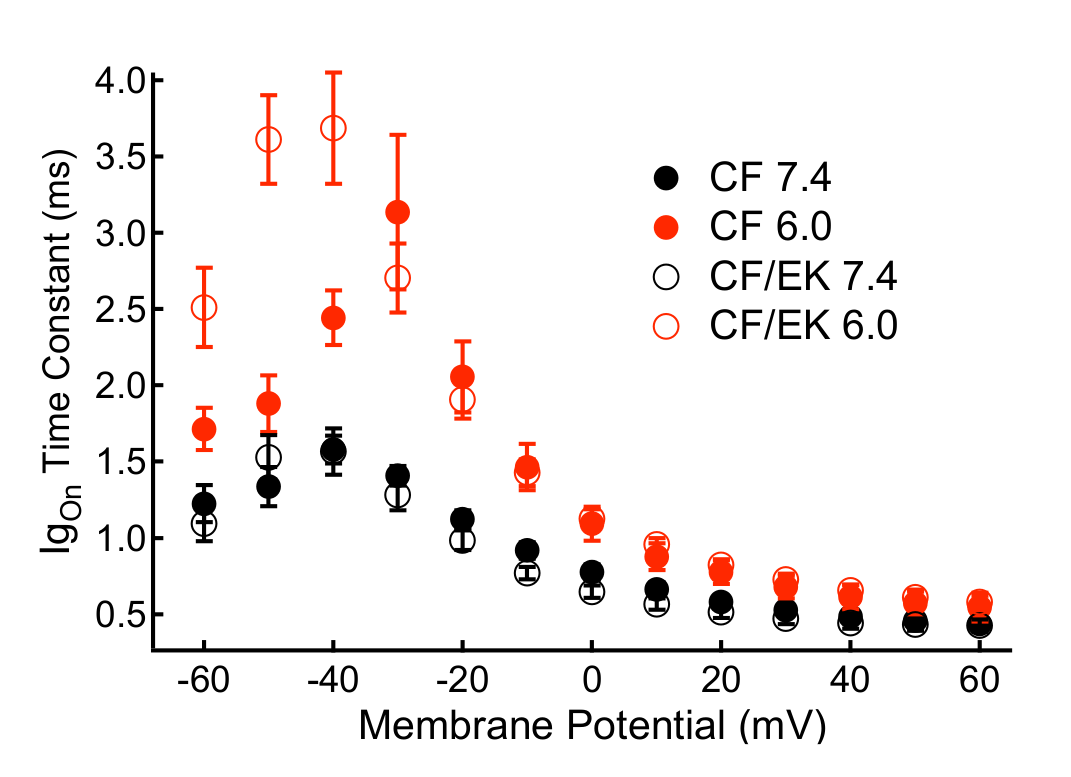

Supplement: S2 Fig — The rate of outward gating charge determined by fitting the decay of outward gating currents with a single exponential is shown for CF and CF/EK channels at pH 7.4 and pH 6.0. (TIFF) [file pone.0184605.s003.tiff]

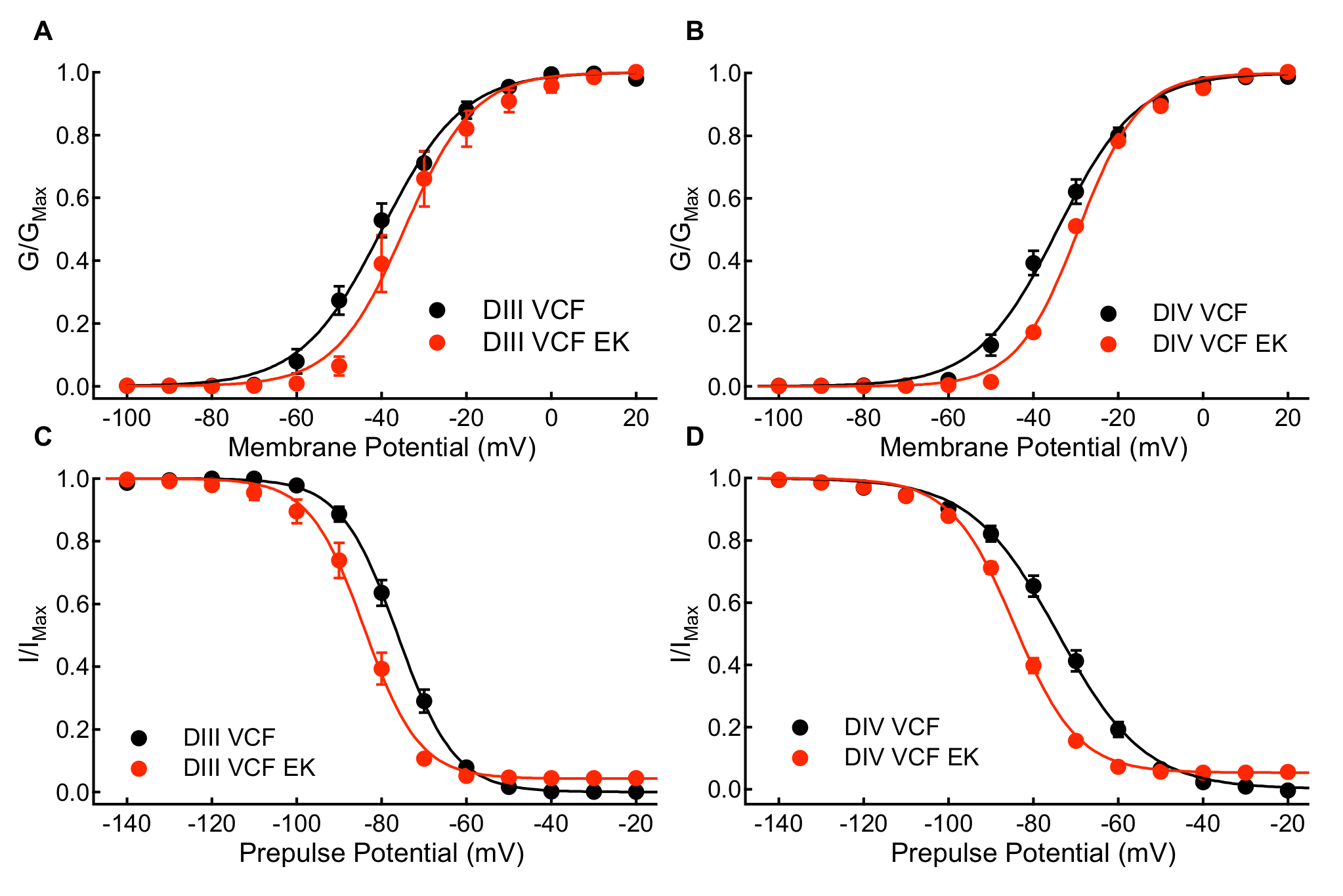

Supplement: S3 Fig — Conductance-voltage relationships for DIII (A) and DIV (B) VCF constructs with and without the E1784K mutant. In both VCF constructs the E1784K mutant depolarizes the conductance-voltage relationship. Steady-state fast inactivation voltage-dependence for DIII (C) and DIV (D) VCF constructs with and without the E1784K mutant. In both VCF constructs the E1784K mutant causes a hyperpolarizing shift in the steady-state fast inactivation voltage-dependence. (TIFF) [file pone.0184605.s004.tiff]

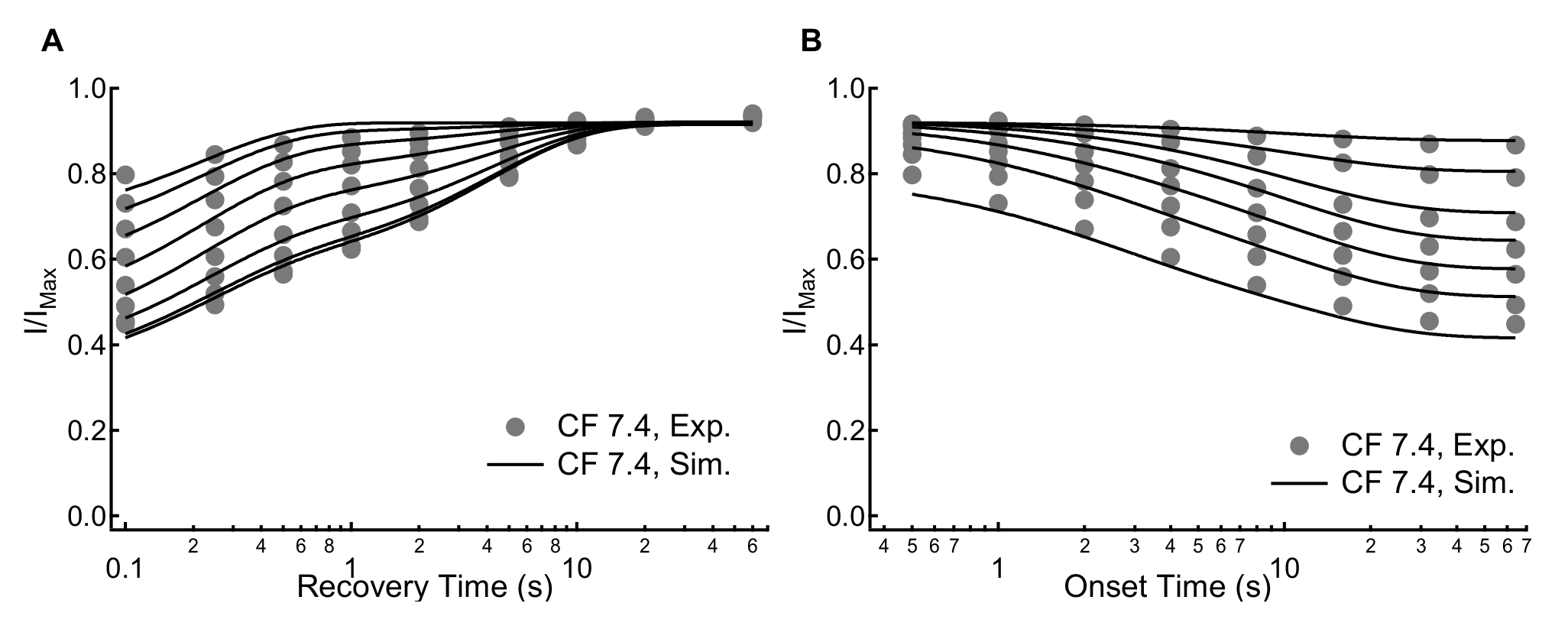

Supplement: S4 Fig — Fits to the simulated slow inactivation recovery time courses at -80 mV after depolarizations to 0 mV ranging between 500 ms (top trace) to 64 s (bottom trace) are overlapped to experimental data (C). Fits to simulated slow inactivation onset time courses at 0 mV with a recovery pulse between 100 ms and 10 s to -80 mV are overlapped with experimental data (D). (TIFF) [file pone.0184605.s005.tiff]
